# A-to-I mRNA editing in bacteria can affect protein sequence, disulfide bond formation, and function

**DOI:** 10.1093/nar/gkaf584

**Published:** 2025-07-02

**Authors:** Liron Didi, Ofir Fargeon, Liam Aspit, Eyal Elias, Dor Braverman, Dganit Melamed, Daniel Keidar-Friedman, Nadav Sorek, Orit Raz, Sharon Ovnat Tamir, Raz Zarivach, Neta Sal-Man, Orna Dahan, Yitzhak Pilpel, Dan Bar Yaacov

**Affiliations:** The Shraga Segal Department of Microbiology, Immunology and Genetics, Faculty of Health Sciences, Ben-Gurion University of the Negev, Beer-Sheva, 8410501 Israel; The Shraga Segal Department of Microbiology, Immunology and Genetics, Faculty of Health Sciences, Ben-Gurion University of the Negev, Beer-Sheva, 8410501 Israel; The Shraga Segal Department of Microbiology, Immunology and Genetics, Faculty of Health Sciences, Ben-Gurion University of the Negev, Beer-Sheva, 8410501 Israel; The Shraga Segal Department of Microbiology, Immunology and Genetics, Faculty of Health Sciences, Ben-Gurion University of the Negev, Beer-Sheva, 8410501 Israel; The Shraga Segal Department of Microbiology, Immunology and Genetics, Faculty of Health Sciences, Ben-Gurion University of the Negev, Beer-Sheva, 8410501 Israel; The Smoler Protein Research Center, Technion Israel Institute of Technology, Haifa, 3200003 Israel; Assuta Ashdod University Hospital, Faculty of Health Sciences, Ben-Gurion University of the Negev, Ashdod, 7747629 Israel; Assuta Ashdod University Hospital, Faculty of Health Sciences, Ben-Gurion University of the Negev, Ashdod, 7747629 Israel; Assuta Ashdod University Hospital, Faculty of Health Sciences, Ben-Gurion University of the Negev, Ashdod, 7747629 Israel; Assuta Ashdod University Hospital, Faculty of Health Sciences, Ben-Gurion University of the Negev, Ashdod, 7747629 Israel; Department of Life Sciences, Ben-Gurion University of the Negev, Beer-Sheva, 8410501 Israel; The Shraga Segal Department of Microbiology, Immunology and Genetics, Faculty of Health Sciences, Ben-Gurion University of the Negev, Beer-Sheva, 8410501 Israel; Department of Molecular Genetics, Weizmann Institute of Science, Rehovot, 7610001 Israel; Department of Molecular Genetics, Weizmann Institute of Science, Rehovot, 7610001 Israel; The Shraga Segal Department of Microbiology, Immunology and Genetics, Faculty of Health Sciences, Ben-Gurion University of the Negev, Beer-Sheva, 8410501 Israel

## Abstract

Adenosine-to-inosine (A-to-I) mRNA editing can alter genetic information at the RNA level and is known to affect protein sequence and function in eukaryotes. However, the ability of A-to-I mRNA editing to recode protein sequences in bacteria was never shown at the protein level. Furthermore, the effect of A-to-I mRNA editing itself—not by A-to-G DNA-mimicking mutations—on protein function was never demonstrated in bacteria. Here, we show at the RNA and protein levels that A-to-I mRNA editing directly recodes a tyrosine to a cysteine residue in the toxin HokB (part of the HokB/*sokB* toxin–antitoxin system in *Escherichia coli*). Consequently, the toxicity of edited HokB increases, inducing bacterial death or early entrance into the stationary phase, depending on its expression level. Furthermore, we demonstrate that *in vivo* disulfide bond formation underlies the effect of A-to-I mRNA editing on HokB function, suggesting that A-to-I mRNA editing constitutes a novel mechanism to regulate disulfide bond formation in bacteria. Finally, we observe that A-to-I mRNA editing of *hokB* is conserved in pathogenic bacteria, supporting functional importance with possible clinical relevance. Our work reveals that A-to-I mRNA editing can constitute a novel mechanism to regulate protein sequence, disulfide bonds, and function in bacteria.

## Graphical Abstract

**Graphical Abstract:**
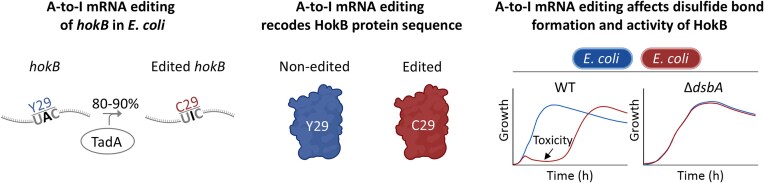

## Introduction

Adenosine-to-inosine (A-to-I) mRNA editing can affect the sequence and function of translated proteins in eukaryotes because their ribosome identifies inosine as guanosine [1–5]. A-to-I mRNA editing occurs in all major multicellular organism (metazoa) groups by enzymes belonging to the adenosine deaminase acting on RNA (ADAR) family [1, 3–11]. A-to-I mRNA editing is essential for preventing an aberrant double-stranded RNA (dsRNA) immune response, crucial for proper neuronal activity, plays a role in cancer progression, affects embryonic development, and diversifies the proteome [10, 12–30]. Furthermore, despite lacking an ADAR homolog, A-to-I mRNA editing was reported in fungi, supporting its occurrence across eukaryotes [31–34]. In contrast, until recently, bacteria were thought to lack A-to-I mRNA editing.

The genome of most bacterial species encodes a tRNA-specific adenosine deaminase (TadA) [35]. Historically, TadA was considered a tRNA-specific editing enzyme, introducing inosine at the anticodon of *tRNA^Arg2^* [35, 36]. Several years ago, we showed that TadA mediates mRNA editing in bacteria (*Escherichia coli*), implicating it in both tRNA and mRNA A-to-I editing [37]. In agreement with our findings, it was recently shown that the homolog of TadA mediates A-to-I mRNA editing in the bacterium *Streptococcus pyogenes* [38] and fungi [39]. Moreover, A-to-I mRNA editing was also shown to occur in the sequence motif of TadA (UACG) in the bacterium *Klebsiella pneumoniae* [40]. In *E. coli*, all 12 sites identified in protein-coding regions are predicted to change tyrosine to a cysteine codon, suggesting that it can recode protein sequence. This prediction is based on the assumption that inosine is identified as guanosine by the bacterial ribosome, similarly to eukaryotes. However, it was never shown, at the protein level, that A-to-I mRNA editing can alter protein sequence in bacteria.

Among the edited sites in *E. coli*, the transcripts of *hokB* have the highest editing level (80%–90%). HokB (Host killing B) belongs to the Hok/*sok* family of type 1 toxin–antitoxin systems [41–43]. HokB is considered a functionally active toxin anchored to the inner membrane by a single alpha helix, with periplasm localization of its C-terminus [42, 44, 45]. HokB was suggested to be expressed in a minority of the bacterial population (0.01%) [46]. HokB is not translated in most bacterial cells because its cognate antitoxin *sokB* (silencing of *hokB*) binds the mRNA of *hokB* and leads to its degradation [42]. HokB expression is induced by the accumulation of the alarmone (p)ppGpp, resulting in a membrane potential collapse and multidrug tolerance [42].

In addition to *hokB*, the genome of the *E. coli* K-12 strain contains four more *hok* genes (*hokA–hokE*). Importantly, we found that editing also occurs in the transcripts of *hokC*, *hokD*, and *hokE*. However, in contrast to the high editing level of *hokB*, the mRNAs encoded by the other *hok* genes are lowly to moderately edited (*hokC*,9%; *hokD*, 6%; and *hokE*, 40% [37]). Furthermore, except *hokB*, all other genome-encoded *hok* homologs are suggested to be inactivated, either by insertion elements (*hokA*, *hokC*, and *hokE*) or by genomic rearrangements, resulting in the absence of components of the canonical toxin–antitoxin systems [41]. Thus, *hokB* currently constitutes the best gene/protein model to study the effect of A-to-I mRNA editing in bacteria.

A-to-I mRNA editing in HokB is predicted to change tyrosine to a cysteine codon at position 29 (Y29C). One way to study the effect of A-to-I editing on protein sequence and function is to use DNA mutations that mimic the predicted editing effect on protein sequence [37, 47]. Indeed, when we expressed HokB with an A-to-G DNA mutation, resulting in Y29→C29 substitution in HokB (A1491986G in NC_00913.3), we found it to induce a strong growth arrest at the population level [37]. However, the effect of A-to-I mRNA editing itself, i.e. when introduced to mRNA molecules and not by DNA mutations mimicking editing, on bacterial protein function and growth was not investigated so far.

Disulfide bonds are covalent bonds between two sulfur atoms of cysteine residues within or between proteins [48]. They can stabilize protein structure and affect their function [48]. Disulfide bonds are formed in oxidizing environments such as in the bacterial periplasm and eukaryotic endoplasmic reticulum [49–51]. In bacteria, disulfide bonds were suggested to occur in most species except in obligate anaerobes or intracellular species [52, 53]. In *E. coli*, disulfide bonds are formed by the thiol-disulfide oxidoreductase DsbA [49]. DsbA is localized to the bacterial periplasm, oxidizing and forming disulfide bonds on proteins as they go through the inner membrane [54]. Subsequently, the protein disulfide isomerase DsbC can break or isomerize (change the location of) DsbA-formed bonds [55].

HokB contains three DNA-encoded cysteine residues at positions 9, 14, and 46. The cysteines at positions 9 (C9) and 14 (C14) are predicted to be embedded in an alpha helix within the inner membrane, while the cysteine at position 46 (C46) is predicted to localize to the periplasm similarly to the mRNA-edited cysteine (C29) [37, 56]. Previously, it was shown that DsbA forms a disulfide bond between two HokB monomers via their C46, which DsbC can break prior to degradation by the protease DegQ [44]. Notably, disulfide bond formation between C46 of two monomers was tested with a non-edited HokB version harboring a tyrosine at position 29 [44]. Under physiological conditions, most transcripts of endogenously expressed *hokB* are edited [37], and thus, most HokB proteins are predicted to have a cysteine at position 29. However, the ability of A-to-I mRNA editing to regulate protein disulfide bond formation in edited HokB or in any bacterial proteins was never shown.

Here, we study the role of A-to-I mRNA editing in HokB and, through it, show for the first time that A-to-I editing can alter protein sequence, disulfide bond formation, and function in bacteria. Thus, A-to-I mRNA editing could constitute a novel RNA-based mechanism to regulate protein activity in bacteria.

## Materials and methods

### Bacterial strains and growth assays

All experiments in this work used the *E. coli* Top10 strains (DH10B-WT, DH10B-Δ*dsbA*, and DH10B-Δ*dsbC*, a generous gift from the lab of Professor Jan Michiels, KU Leuven), except for the pathogenic bacterial species [enterohemorrhagic *E. coli* (O157:H7 EDL933), enteropathogenic *E. coli* (O127:H6 strain E2348/69), uropathogenic *E. coli* that was isolated from a patient suffering from urinary tract infection, and *Shigella sonnei* (ATCC 25931)]. Cultures were grown at 37°C for 20–24 h in LB medium (5 g yeast extract, 10 g NaCl, and 10 g tryptone in 1 l of double-distilled water) supplemented with 100 μg/ml ampicillin in New Brunswick Innova^®^ 42R shaker incubator at 200 rpm. Cultures were back diluted in a 1:100 ratio and dispensed on 96-well plates (Corning Costar) containing 150 μl of LB medium supplemented with 100 μg/ml ampicillin and 0.2% arabinose or 0.0002% arabinose for inducing HokB expression, and 1mM of Isopropyl β-D-1-thiogalactopyranoside (IPTG) for inducing GFP or GFP-TadA expression (final concentrations). Wells were measured every 30 min for optical density at OD_600_ and mCherry fluorescence for 24 h in a Tecan-Spark plate reader (orbital shaking, 180 rpm, 3 mm amplitude). For each strain, the mean of either OD or mCherry was calculated by averaging 21 wells. The 96-well plate was divided as follows: 12 wells were blank control containing only the relevant media and the remaining 84 wells were divided between the bacterial stains as indicated in the different figures and were dispersed throughout the plate to control for geographical effects on growth. OD measurements were taken at 600 nm. Excitation wavelengths of 485 (±20) and 560 (±20) nm with emission wavelengths at 535 (±25) and 620 (±20) nm were used for GFP and mCherry measurements, respectively. The figures throughout the work represent the average of three independent experiments conducted on different days with cultures from different colonies (unless otherwise mentioned).

### Plasmid construction

As endogenous translation of HokB is blocked by the antitoxin *sokB* in almost all bacterial cells in culture, we expressed HokB (fused to mCherry) from an inducible plasmid system, as others before us [37, 42]. Notably, the plasmid-borne mRNA of *hokB* contains the original coding sequence as found on the chromosome (NC_000913.3), however, without the binding site for *sokB* antitoxin, allowing HokB translation [37, 42].

The plasmids encoding for GFP-TadA and GFP only were created by NEBuilder^®^ HiFi DNA Assembly. To construct pME6032-GFP-TadA, we used primers 1 and 2 for the plasmid backbone, and primers 3 and 4 for the *tadA* gene (amplified from the chromosome of *E. coli* NC_000913.3).

To construct pME6032-GFP, we used primers 5 and 6 for the plasmid backbone, and primers 7 and 8 for the *gfp* gene.

To examine the importance of other cysteine residues in HokB, we mutated cysteine codons at different sites to serine in the plasmids expressing the different versions of HokB. We amplify the entire plasmid using high-fidelity Kapa DNA polymerase (Roche) with back-to-back primer pairs containing a single-point mutation at a desired location. We used primers 9 and 10 to insert the C9S substitution, primers 11 and 12 to insert the C14S substitution, and primers 13 and 14 to insert the C46S substitution. Following polymerase chain reaction (PCR), products were visualized on a 0.5% agarose gel and cleaned using the DNA Clean & Concentrator Kit (Zymo). Next, products were phosphorylated by T4 Polynucleotide Kinase (NEB), ligated by T4 DNA ligase (NEB), and treated with DPN1 (NEB) to eliminate any original plasmid remains that were used as a template for the PCR reaction.

The plasmids encoding for DsbA and DsbC were created by NEBuilder^®^ HiFi DNA Assembly. To construct pME6032-DsbA, we used primers 15 and 16 for the plasmid backbone, and primers 17 and 18 for the *dsbA* gene (amplified from the chromosome of *E. coli* NC_000913.3). To construct pME6032-DsbC, we used primers 19 and 20 for the plasmid backbone, and primers 21 and 22 for the *dsbC* gene (amplified from the chromosome of *E. coli* NC_000913.3). All primers are found in Supplementary Table S1.

All enzymatic reactions were done according to the manufacturer’s instructions. The new versions of the plasmid were transformed into Top10 (DH10B) *E. coli*.

### cDNA synthesis and reverse transcription quantitative polymerase chain reaction

RNA was purified by GeneJET RNA Purification Kit (Thermo Fisher Scientific, K0731) after 3 h and 30 min of growth by our laboratory. RNA was eluted using ultra-pure water (PCR grade; Tamar-UPW-500). RNA samples were treated with DNase I (RNase-free) (NEB-M0303L) for 20 min at 37°C and cleaned using RNA Clean & Concentrator™-5 w/ Zymo-Spin™ IC Columns (Zymo-ZR-R1015) [samples were eluted with ultra-pure water (PCR grade; Tamar-UPW-500)]. RNA samples were frozen at −80°C immediately after extraction. To synthesize cDNA, 500 ng of total RNA (measured by DeNovix DS-11 FX Spectrophotometers/Fluorometers) from *E. coli* strains was primed by us with random hexamers and reverse transcribed with the GoScript Reverse Transcription Mix Kit (Promega-A2801) following the manufacturer’s protocol in a total volume of 20 µl. cDNA samples were frozen at −20°C before qRT-PCR (reverse transcription quantitative polymerase chain reaction) performance. For all samples, cDNA was PCR-amplified using LightCycler^®^ 480 Instrument II (Roche) with the LightCycler 480 SYBR Green I Master [Roche, 4707516001; ready-to-use hot start PCR mix—contains faststart Taq DNA polymerase, reaction buffer, dNTP mix (with dUTP instead of dTTP), SYBR Green I dye, and MgCl_2_] in 384-multiwell plate (Roche, 04729749001) in a total volume of 6 µl. Primers were used at a final concentration of 0.5 µM. The transcript of *rpoA* was used as an internal control to normalize the data. The results represent four biological replicates per sample, each with three technical replicates. We used primers 27 and 28 produced by Merck to amplify the cDNA of *hokB*, the length of the amplicon was 108 bp, and primers 29 and 30 produced by Merck (formerly Sigma–Aldrich) to amplify the cDNA of *rpoA*. The length of the amplicon was 114 bp. *T*_m_ of the reaction was 60°C.

### RNA purification and amplicon sequencing of plasmid-borne *hokB*

RNA was purified by GeneJET RNA Purification Kit (Thermo Fisher Scientific) after 2 h and 15 min, before the toxic effect of edited HokB was observed (a decrease in OD when co-expressing HokB and TadA) to ensure that bacteria are not lysing and affecting the analysis. When only the different version of HokB were expressed, RNA was extracted after 3 h and 30 min of growth to allow the culture to reach the mid-logarithmic phase. Libraries to examine RNA editing levels of *hokB* versions expressed from plasmids were constructed as follows: first, cDNA was synthesized as described. Next, we used primers 23 and 24 to target the *hokB* transcript and add sequence tails that match Illumina’s adapters. Notably, primer 23 is localized to the linker sequence between *mcherry* and *hokB* (only three bases overlap the sequence of *hokB*), while primer 24 is localized to the 3′ untranslated region of the transcript with a sequence specific to the plasmid backbone. Thus, the primers can only amplify the cDNA of the *hokB* transcript transcribed from the plasmid. PCR conditions were as follows: initial denaturation for 3 min at 95°C; followed by 10 cycles of (i) denaturation for 20 s at 98°C, (ii) annealing for 15 s at 55°C, and (iii) elongation for 10 s at 72°C; at the end of the cycles, a final elongation step for 60 s at 72°C was added. Then, a second PCR was carried out with primers 25 and 26 using the first PCR product as a template. This PCR was used to add oligos/adapters for the Illumina run with dual index. PCR conditions were as follows: initial denaturation for 3 min at 95°C; followed by 30 cycles of (i) denaturation for 20 s at 98°C, (ii) annealing for 15 s at 60°C, and (iii) elongation for 10 s at 72°C; at the end of the cycles, a final elongation step for 60 s at 72°C was added. We used KAPA HiFi HotStart ReadyMix (Roche, #07958935001) for the PCR reactions. Libraries were sequenced using 150-nt paired-end reads on the NovaSeq X or NextSeq 550 platforms (Illumina).

### RNA editing analysis of the amplicon sequencing data

To identify and quantify RNA editing levels, we used CLC Genomics Workbench (QIAGEN Bioinformatics) for all steps of the analysis.

First, we used the “Trim Reads” tool to ensure the high quality of the reads by trimming the reads according to length and quality scores using the following parameters: quality limit = 0.05; trim ambiguous nucleotides = yes; maximum number of ambiguities = 2; automatic read-through adapter trimming = yes; and minimum length = 50.

Next, we used the “Map Reads to Reference” tool to map the reads to the *E. coli* reference genome (NC_000913.3) with the following parameters: masking mode = no masking; match score = 1; mismatch cost = 2; cost of insertions and deletions = linear gap cost; insertion cost = 3; deletion cost = 3; length fraction = 0.95; similarity fraction = 0.95; global alignment = no; auto-detect paired distances = yes; and non-specific match handling = ignore.

Next, we used the “Basic Variant Detection” tool for variant calling using the following parameters: minimum coverage = 20; minimum count = 3; minimum frequency (%) = 1.0; base quality filter = yes; neighborhood radius = 2; minimum central quality = 30; and minimum neighborhood quality = 30. Finally, we focused on the edited site of *hokB* in the plasmid expressing HokB and non-edited HokB.

### Membrane fraction enrichment for western blot analysis and protein mass spectrometry

*Escherichia coli* co-overexpressing HokB and GFP or GFP-TadA were grown at 37°C for 2 h and 15 min in LB medium supplemented with 100 μg/ml ampicillin (Tivan Biotech AMP-5G), 10 μg/ml tetracycline (GoldBio, T-10125), 0.2% arabinose (l-(+)-arabinose HPLC 99%, Tivan Biotech, ARA100G), and 1 mM IPTG (GoldBio, I2481C5) in New Brunswick Innova^®^ 42R shaker incubator at 200 rpm. One milliliter of cells were centrifuged at 4°C, 3000 × *g* for 10 min. The pellet was resuspended with 1 ml cold 1× PBS (BioLab 1623237500) and centrifuged again at 4°C, 10 000 × *g* for 1 min. The pellet was resuspended in 500 μl lysozyme buffer: 150 mM NaCl (BioLab, 001903029100), 30 mM Tris–HCL (pH 8) (BioLab, 2085232300), and 10 mM EDTA (pH 8) (BioLab, 9012237500), supplemented with 1 mg/ml lysozyme (GoldBio, L-040-5), protease inhibitor (Sigma Protease Inhibitor Cocktail Set III, EDTA-Free; 539134-1ML) diluted 1:200, and 1 mM dithiothreitol (DTT; Tivan Biotech, DTT005), and frozen at −20°C overnight. Cells were thawed for 5 min at 25°C thermal block without shaking and later incubated for 10 min at 37°C in New Brunswick Innova^®^ 42R shaker incubator at 200 rpm. Each sample was treated with 3 ml DNase solution: 10×DNaseI buffer (NEB, B0303S) and DNaseI 2000 units/ml (NEB, M0303L) according to protocol recommendation, 1 mM DTT, and 1 mM phenylmethylsulfonyl fluoride (Sigma, P7626-1G) and incubated for 10 min at 37°C in New Brunswick Innova^®^ 42R shaker incubator at 200 rpm. Samples were transferred to ice for 5 min. Membrane protein fraction was collected by centrifugation at 4°C, 20 000 × *g*, for 20 min. The pellet was resuspended in 50 μl cold 1×PBS.

### Western blot analysis

We grew strains at 37°C for 2 h and 30 min in LB medium supplemented with 100 μg/ml ampicillin. Next, arabinose was added (to a final concentration of 0.2%) and bacteria were placed back into the incubator for 90 s. This short induction time was used because of the toxicity of expressed C29 containing HokB that leads to rapid lysis of *E. coli*. Bacteria were lysed as described in the “Membrane fraction enrichment for western blot analysis and protein mass spectrometry” section. Protein quantification was determined using QPRO—BCA Kit Standard (Cyanagen, 9470BCASTAND500) according to the manufacturer’s protocol. For the standard curve, we used BSA (bovine serum albumin) ampules (Thermo Fisher Scientific, TS-23209). We normalized the samples in each replicate to the sample with the lowest protein concentration. About 15–30 μg of membrane fraction protein extract was loaded on 12% sodium dodecyl sulfate–polyacrylamide gel electrophoresis (SDS–PAGE) (same amount of protein in each gel). Samples ran on gel for 10 min at 100 V and another 50 min at 200 V. Blotting was done into the Trans-Blot Turbo Mini 0.2 μm Nitrocellulose Transfer (Bio-Rad, 1704158) using Trans-Blot Turbo-Transfer System (Bio-Rad) with the preset manufacturer’s protocol for high molecular weight proteins. The blots were blocked with 5% nonfat dry milk in TBST (Tris-buffered saline, 0.05% Tween) (skim milk powder; Millipore, 70166-500G) (TTBS; biolab, 2089232300) and incubated with the primary antibody anti-RFP (mCherry) (purified IgG/rabbit anti-RFP pAb; MBL-PM005) at 4°C, overnight. Membranes were incubated for 1 h at 4°C with an HRP-conjugated secondary antibody (Peroxidase AffiniPure™ Goat Anti-Rabbit IgG (H+L); Jackson, 111–035-003). Rabbit-anti-OmpA was used as internal control for protein quantity and membrane fraction enrichment [57]. The membrane was visualized using ECL (WESTAR ANTARES, 9470ANTARES250) on the Azure 400 Visible Fluorescent Imager (Azure Biosystems, AZI400-01).

### Proteolysis

Twenty micrograms of proteins from the membrane fraction of *E. coli* co-overexpressing HokB and GFP or GFP-TadA was loaded on 4%–15% gradient SDS–PAGE in three replicates. The proteins in the gel slices that contain the expected size of the mCherry-HokB proteins (∼33 kDa) were reduced with 3 mM DTT (60°C for 30 min), modified with 10 mM iodoacetamide in 100 mM ammonium bicarbonate (in the dark, at room temperature for 30 min), and digested in 10% acetonitrile and 10 mM ammonium bicarbonate with modified trypsin (Promega) at a 1:10 enzyme-to-substrate ratio, overnight at 37°C. An additional second digestion with trypsin was done for 4 h at 37°C. The tryptic peptides were desalted using C18 tips (homemade stage tips), dried, and resuspended in 0.1% formic acid.

### Mass spectrometry analysis

The resulting peptides were analyzed by LC–MS/MS using Exploris 480 mass spectrometer (Thermo) fitted with a capillary HPLC (Evosep).

The peptides were loaded onto a 15-cm, ID 150-μm, 1.9-μm Endurance Column EV1137 (Evosep). The peptides were eluted with the built-in 15 SPD (88 min) method.

Mass spectrometry was performed in a positive mode using repetitively full MS scan (*m*/*z* 350–1200) followed by high energy collision dissociation in two separate scan events. First event: the 20 most dominant ions (>1 charges) were selected from the full MS scan; a dynamic exclusion list was enabled with an exclusion duration of 30 s. A second scan event: the 20 most dominant ions (>1 charges) were selected from a mass list without dynamic exclusion.

### Data analysis

The mass spectrometry data were analyzed using Protein Discoverer 2.4 (Thermo) using Sequest search engine, searching against the *Escherichia coli*_strain K12 proteome (UP000000625) from the UniProt database (downloaded in May 2023; 4403 entries) and specific sequences of edited proteins (with the different amino acids), with mass tolerance of 20 ppm for the precursor masses and 0.02 Da for the fragment ions. Oxidation on methionine and protein N-terminus acetylation were accepted as variable modifications, and carbamidomethyl on cysteine was accepted as static modifications. Minimal peptide length was set to six amino acids and a maximum of two miscleavages were allowed. The data were quantified by label-free analysis using the same software. Peptide-level FDRs were filtered to 1% using the target-decoy strategy.

### CFU counts

Cultures were grown at 37°C for 20–24 h in LB medium, in New Brunswick Innova^®^ 42R shaker incubator at 200 rpm. Cultures were back diluted in a 1:100 ratio in a tube containing LB medium supplemented with 100 μg/ml ampicillin and grown in a Tecan-Spark plate reader (orbital shaking; 180 rpm; 3 mm amplitude) or Biotek Synergy H1 (orbital shaking; 180 rpm; 2 mm amplitude). After 4 h of growth, samples from each strain were diluted in a factor of 10^−6^ and 50–100 μl was plated on LB agar plates supplemented with 100 μg/ml ampicillin. In parallel, arabinose was added to a final concentration of 0.2% to the wells for an additional 1 h of growth. After a total of 5 h from the beginning of growth, samples from each strain were diluted in a factor of 10^−4^ to 10^−6^ and 50–100 μl was plated on LB agar plates supplemented with 100 μg/ml ampicillin. Colonies were manually counted and normalized to a 1 ml volume of bacteria.

### PCR of DNA and cDNA samples of *E. coli* and *S. sonnei*

RNA and DNA were extracted at the mid-logarithmic phase (OD_600_ = 0.5–0.8) as described above. PCR was conducted with primers 31 and 32 that match a conserved sequence in *hokB* of *E. coli* and *S. sonnei*.

### RNA secondary structure prediction

Prediction was made using 25 upstream and downstream bases of the edited adenosine in the mRNA of *hokB* as we did before using RNAfold [37, 58] or with the entire coding sequence of HokB shown.

## Results

### A-to-I mRNA editing can affect protein sequence and function in bacteria

**Figure 1.**
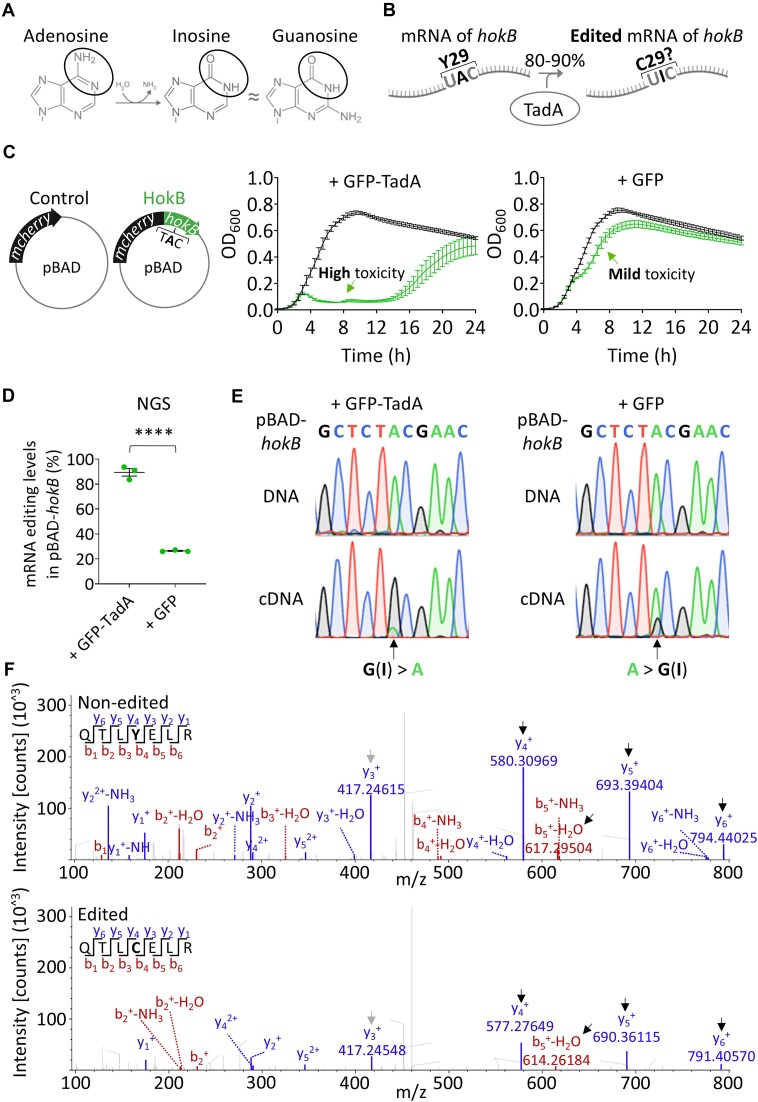
A-to-I mRNA editing can affect protein sequence and function in bacteria as evidenced by the case of HokB. (**A**) Adenosine is deaminated to inosine, which is similar to guanosine in its base-pair properties. (**B**) A-to-I mRNA editing by TadA in *E. coli* occurs in 80%–90% of endogenously expressed *hokB* transcripts at the logarithmic phase, and is assumed to recode a tyrosine to a cysteine codon at position 29 of HokB. (**C**) Growth analysis of *E. coli* (Top10-DH10B) co-expressing mCherry (control, black) or mCherry-HokB (green), with either GFP-TadA (left panel) or GFP (right panel). The mean and standard error of three biological replicates conducted on different days (*N* = 3), each with 21 technical replicates, are shown. The expression of mCherry and HokB was induced from the beginning of the experiment (time point “0”) with 0.2% arabinose from a pBAD vector. Expression of GFP-TadA or GFP was induced with 1 mM IPTG from a pME6032 vector. (**D**) A-to-I mRNA editing identification by next-generation sequencing (NGS; Illumina; amplicon-seq) of plasmid-borne *hokB* RNA (cDNA) co-overexpressed with GFP-TadA or with GFP alone. Minimum observed reads coverage per sample that passed our quality filters ≥17 515 391. Statistical analysis was conducted using Student’s *t*-test; *P*-value <.0001 (****). (**E**) A-to-I mRNA editing validation by Sanger sequencing of plasmid-borne DNA and RNA (cDNA) of *hokB* when co-overexpressed with GFP-TadA or with GFP. The sequence above the chromatograms represents the gene (DNA) sequence. A black arrow marks the double peak of A and G(I) that was observed only in the cDNA (complementary DNA) samples. Note that the G(I) peak is higher than the A peak when overexpressing GFP-TadA and vice versa when overexpressing only GFP. (**F**) MS/MS spectrum of non-edited (Y29; top) and edited (C29; bottom) HokB peptides found in strains co-overexpressing HokB with GFP (top) or GFP-TadA (bottom). Black arrows mark identified peptides and their mass in the MS/MS spectra that show a mass shift corresponding to tyrosine or cysteine at the edited site. The gray arrow marks an example of a peptide and its mass in the MS/MS spectra that does not include the edited site (same mass). All peptides were discovered with false discovery rate (FDR) ≤ 0.01. The peaks weight, font size, and axis were adjusted from the original figure for better visualization and comparison. A comprehensive mass distribution and the original MS/MS spectra and data can be found in Supplementary Tables S2–S4.

As mentioned in the introduction, we previously used A-to-G DNA mutations to mimic the effect of A-to-I mRNA editing, assuming that inosine is identified as guanosine by the bacterial ribosome (Fig. 1A and B). However, the effect of A-to-I mRNA editing itself—at the RNA level and not by A-to-G DNA-mimicking mutations—on protein sequence and function in bacteria was never examined. To test the effect of RNA editing in bacteria, we co-overexpressed HokB and TadA (from two different plasmids). We used this two-plasmid system because we suspected that endogenous TadA expression is insufficient to edit the plasmid-borne *hokB* transcript to similar editing levels as in endogenously expressed *hokB*. Importantly, we tagged HokB with mCherry and TadA with GFP to validate their expression and used *E. coli* expressing mCherry and GFP only as controls (Fig. 1C and Supplementary Fig. S1A–D).

We observed that *E. coli* co-overexpressing HokB and TadA displayed strong growth inhibition at the population level (Fig. 1C and Supplementary Fig. S1A and B). In contrast, overexpressing HokB in the presence of a control plasmid lacking TadA (expressing GFP only) resulted in mild toxicity (Fig. 1C and Supplementary Fig. S1C and D).

We interpret the delayed growth in the strain co-overexpressing HokB and TadA as caused by mutants resistant to HokB or mutants that found a way to shut down HokB or TadA expression. Thus, we repeated the growth assays with colonies isolated at the end of the growth experiment after exposure to highly edited HokB. Indeed, we observed that bacteria derived from these colonies grew normally in the presence of HokB expression, as evident from the mCherry signal observed in all three selected colonies (Supplementary Fig. S2). We also observed that some colonies did not express TadA from the plasmid, likely preventing high editing in *hokB* and subsequent toxicity. In another colony, we observed a similar GFP-TadA signal as in the original experiment (Fig. 1C and Supplementary Fig. S2), suggesting another solution that underlies the resistance to edited HokB. To conclude, the delayed growth observed upon expression of mainly edited HokB is likely the result of genetic mutations either preventing TadA expression from the plasmid or conferring resistance in a different form.

Next, we aimed to examine the state of A-to-I mRNA editing in the plasmid-borne *hokB* transcript. Examination of the RNA sequence by NGS and Sanger sequencing revealed that the plasmid-borne *hokB* transcript was highly edited when co-overexpressed with TadA (89.4% ± 5.2%; mean ± SD) (Fig. 1D and E), resembling the endogenous editing levels of *hokB* when expressed from the chromosome (80–90% edited [37]). In contrast, we observed only a moderate level of editing in the control (26.4% ± 0.7%; mean ± SD) (Fig. 1D and E), probably because the endogenous TadA expression is insufficient to edit the plasmid-borne *hokB* transcript.

Finally, we performed mass spectrometry and observed at the protein level that A-to-I editing introduces a cysteine at position 29 of HokB (Fig. 1F, Supplementary Fig. S1E and F, and Supplementary Tables S2–S4). Collectively, our results support that A-to-I mRNA editing can affect protein sequence and function in bacteria, as shown in the case of HokB.

### The toxicity of edited HokB requires mRNA-edited and DNA-encoded cysteines

**Figure 2.**
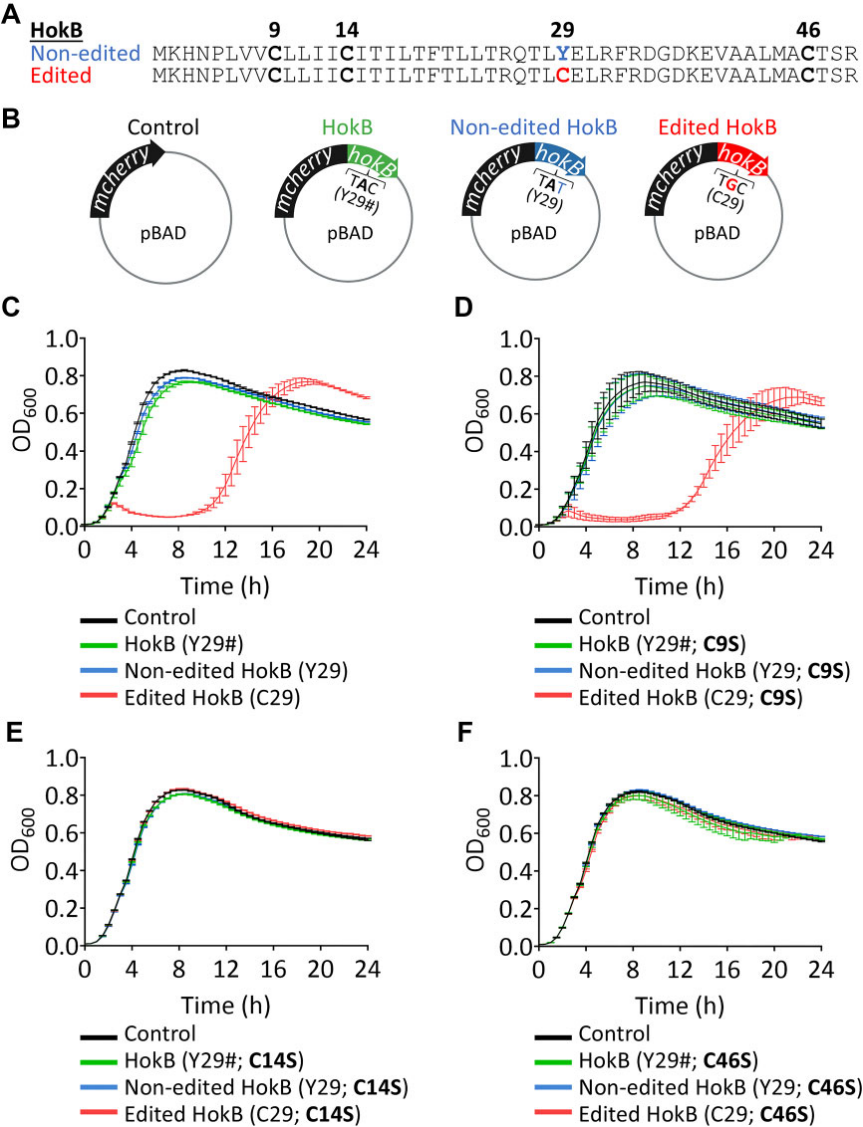
DNA-encoded cysteine residues are essential for the toxicity of edited HokB. (**A**) The protein sequence of non-edited and edited HokB according to their respective transcript. DNA-coded cysteines are shown in bold. (**B**) A description of the different plasmids containing different versions of HokB used in the growth assay is presented. (**C**) Growth analysis of *E. coli* (Top10-DH10B) WT strain expressing the HokB (Y29#, green), non-edited HokB (Y29, blue), and edited HokB (C29, red) fused to mCherry reporter protein (N-terminus) from the plasmid shown in panel (B). As a reference control, a plasmid harboring only mCherry was used (black). As previously reported [37], when highly expressed, edited HokB (C29) induces the highest level of toxicity. (**D**) Growth analysis as in panel (C), with all three versions of HokB having the C9S substitution. (**E**) Growth analysis as in panel (C), with all three versions of HokB having the C14S substitution. (**F**) Growth analysis as in panel (C), with all three versions of HokB having the C46S substitution. In all growth experiments, protein expression was induced from the beginning of the experiment (time point “0”) with 0.2% arabinose from a pBAD vector. The mean and standard error of three biological replicates conducted on different days (*N* = 3), each with 21 technical replicates, are shown.

What molecular effect or mechanism underlies the change in the toxicity of HokB when containing the mRNA editing-dependent cysteine at position 29? As mentioned in the introduction, HokB contains three DNA-encoded cysteine residues at positions 9, 14, and 46 (Fig. 2A). Because TadA introduces a cysteine codon at position 29 of HokB, it could be involved in a disulfide bond formation. If disulfide bonds are involved in the toxicity of edited HokB, then the presence of other cysteine residues should be essential for the toxicity of HokB. To examine the importance of other cysteines for the toxicity of HokB, we expressed different versions of HokB from our inducible plasmid system [37, 42, 44] (Fig. 2B–F). First, as a positive control, we used our plasmids and expressed three different versions of HokB (fused to mCherry): (i) a version that has the original gene sequence of *hokB* with an editable tyrosine codon at position 29 (TAC, designated as Y29# and termed HokB when referring to its protein product, also shown in Fig. 1C); (ii) a version with a non-editable tyrosine codon in the plasmid-encoded *hokB* that disrupts the motif of TadA, and prevents editing without affecting the predicted secondary structure of the transcript (TAT, designated as Y29 and termed “non-edited HokB” when referring to its protein product; Supplementary Fig. S3A and B); and (iii) the version that mimics constitutive editing as it has a cysteine codon in the plasmid-encoded *hokB* (TGC, designated with C29 and termed “edited HokB” when referring to its protein product).

As previously shown, high expression of edited HokB induced enhanced toxicity, while expression of HokB or non-edited HokB induced a mild reduction in growth compared to the control (Fig. 2C and Supplementary Fig. S3C) [37]. The resemblance in phenotype between strains expressing HokB and non-edited HokB is explained by the low editing level of the plasmid-borne editable *hokB* transcript (Supplementary Fig. S3A). Thus, in most cases, HokB encoded by the editable plasmid version will have a tyrosine at position 29.

Next, we introduced a single point mutation to recode a single cysteine to serine at different positions of HokB (C9S, C14S, and C46S). The C-to-S substitutions at positions 14 (C14S) and 46 (C46S), but not 9 (C9S), abolished the editing-dependent toxicity (Fig. 2D–F and Supplementary Fig. S3D–F). Thus, having a cysteine at positions 14 and 46 (but not at position 9) is essential for the toxicity of position 29-edited HokB. These results agree with sequence conservation between Hok homologs encoded in *E. coli* [37, 41]. Specifically, while position 9 is not conserved, position 14 harbors a cysteine across Hok homologs, attesting to its possible importance for the activity of Hok proteins. Moreover, position 46 harbors a cysteine in Hok homologs, either DNA-coded (HokA and HokB) or RNA-editing-coded (HokC, HokD, and HokE), suggesting that the cysteine at position 46 is important [37]. Thus, editing-dependent C29 might interact with C14 or C46 via disulfide bond formation to elicit the observed toxic response.

### *In vivo* disulfide bond formation is essential for the toxicity of the edited HokB

**Figure 3.**
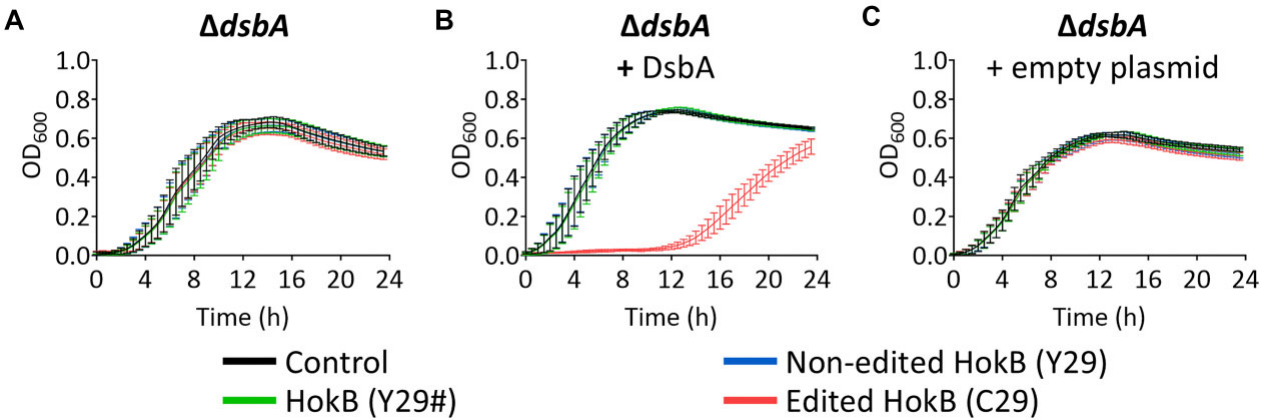
*In vivo* disulfide bond formation is essential for the toxicity of the edited HokB. (**A**) Growth analysis of an *E. coli* Δ*dsbA* strain that expresses one of three versions of HokB, fused to mCherry from an inducible plasmid. As a reference control, we used a plasmid encoding only mCherry. (**B**) Growth analysis as in panel (A), but with overexpressing DsbA from a second plasmid (pME6032). (**C**) Growth analysis, as in panel (B), using an empty plasmid (pME6032 with no *dsbA* insert).

The abolished toxicity of edited HokB when mutated to have either C14S or C46S suggests that the editing-dependent cysteine (C29) interacts with either C14 or C46 via a disulfide bond. If this is true, DsbA activity will be essential for the observed toxicity upon expressing edited HokB. Thus, we expressed the different versions of HokB in a Δ*dsbA* strain, incapable of forming disulfide bonds [44]. Indeed, the toxicity of edited HokB is abolished in a Δ*dsbA* strain (Fig. 3A and Supplementary Fig. S4A). Moreover, expressing DsbA from a second plasmid in the Δ*dsbA* strain restored the toxicity of edited HokB (Fig. 3B and C, and Supplementary Fig. S4B and C).

As mentioned in the introduction, DsbC can break DsbA-formed disulfide bonds [44, 55]. Thus, we next tested whether the activity of DsbC could affect the toxicity of edited HokB. We observed that lack of DsbC (in a Δ*dsbC* strain) or overexpressing DsbC did not affect edited HokB-dependent toxicity (Supplementary Fig. S5). Interestingly, overexpressing DsbC resulted in reduced growth across all tested strains, likely due to excess DsbC activity that affects disulfide bonds across the bacterial proteome.

We conclude that *in vivo* disulfide bond formation is essential for the toxicity of edited HokB.

### A-to-I RNA editing likely mediates disulfide bond formation within HokB monomers

According to our analyses thus far (Figs 2 and 3), the editing-dependent disulfide bond could be formed between or within HokB monomers, bonding between C29 and C14 or C46. To tackle these open questions, we performed western blot analysis on the membrane fraction of *E. coli* harboring the different versions of HokB with or without the cysteine-to-serine substitutions (C9S, C14S, and C46S) as shown in Fig. 2C–F.

**Figure 4.**
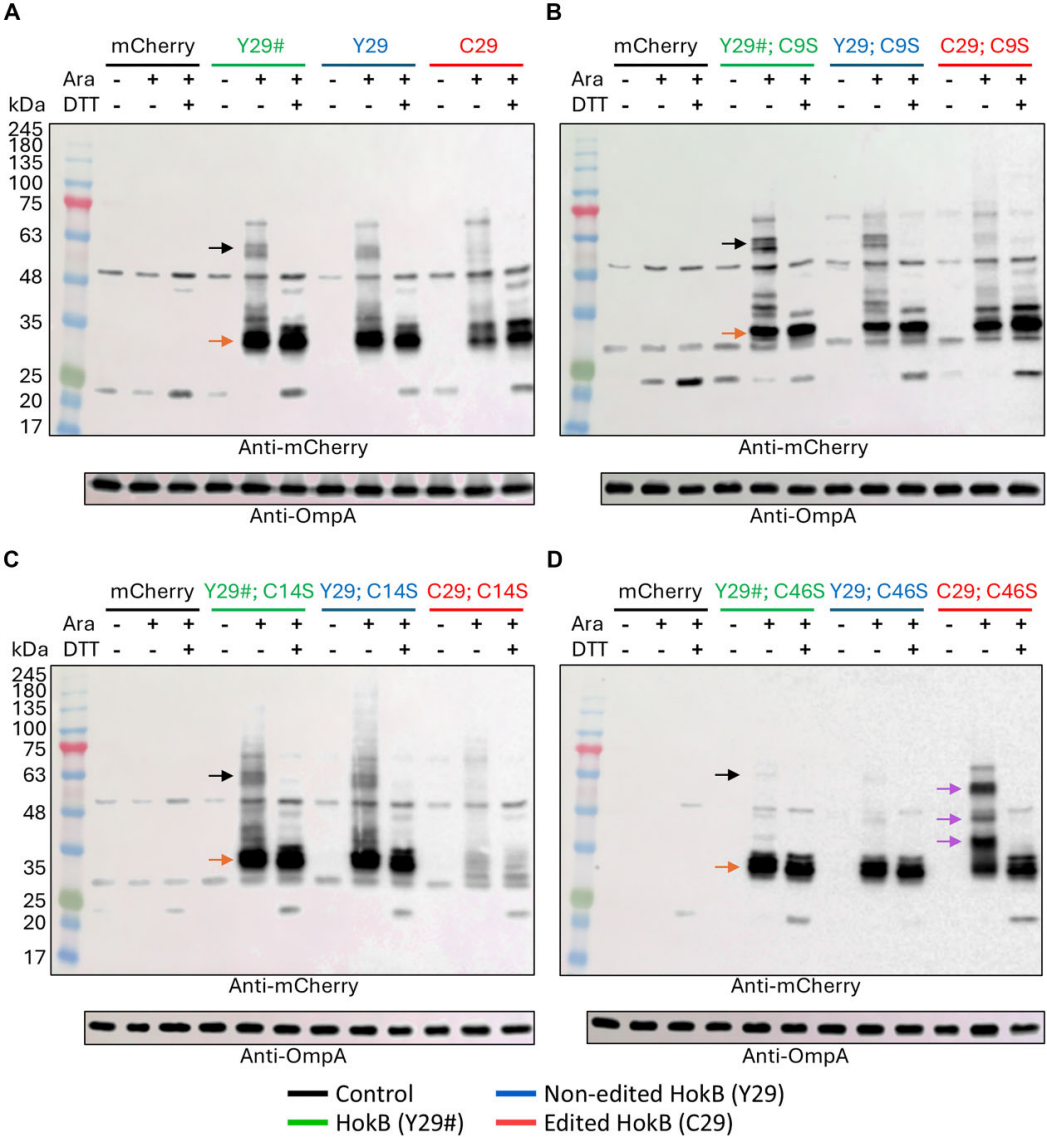
Western blot analysis supports that A-to-I mRNA editing mediates an intramolecular disulfide bond between C29 and C46 in HokB. (**A**) Western blot of membrane enriched protein fraction of *E. coli* (Top10-DH10B) WT strain expressing either mCherry only (control; black) or the HokB (Y29#, green), non-edited HokB (Y29, blue), and edited HokB (C29, red) fused to mCherry (N-terminus) from the plasmid shown in Fig. 2B. (**B**) Same as panel (A) but with the C9S substitution in the different expressed HokB versions. (**C**) Same as panel (A) but with the C14S substitution in the different expressed HokB versions. (**D**) Same as panel (A) but with the C46S substitution in the different expressed HokB versions.

First, our analysis revealed that expressing HokB (Y29#), non-edited HokB (Y29), and edited HokB (C29) resulted in multiple bands, where the strongest band was observed close to the estimated size of mCherry-HokB (≈30–31 kDa) (Fig. 4A). We also detected bands in the size of ≈60 kDa that disappeared upon treatment with the reducing agent DTT (Fig. 4A). Importantly, these bands were not observed in our control, expressing only mCherry. Thus, these bands possibly represent the previously suggested disulfide bond-dependent HokB dimer [44], or interaction between HokB and other membrane proteins via a disulfide bond. Notably, the intensity of these bands was weaker to non-visible when edited HokB was expressed, suggesting that the presence of C29 hinder their appearance (Fig. 4A and Supplementary Fig. S6A).

Second, when expressing HokB (Y29#), non-edited HokB (Y29), and edited HokB (C29) with a cysteine or a serine at position 9 of HokB, similar blot was obtained as when C9 was present (Fig. 4A and B, and Supplementary Fig. S6A-C). This makes sense because expressing the different versions of HokB with a cysteine or a serine at position 9 yielded similar growth phenotypes (Fig. 2C and D).

Third, we observed that in the strain expressing the C14S version of edited HokB, the signals from the main protein band, representing the edited HokB monomer, and the other observed bands were very weak (Fig. 4C and Supplementary Fig. S6D). Thus, the observed rescue of growth observed when C14 is mutated into S14 is the result of decreased abundance of edited HokB in the membrane fraction.

Fourth, when C46 was mutated into S46, the 60-kDa bands almost completely disappeared when the C46S versions of the HokB (Y29#) and non-edited HokB (Y29) were expressed (Fig. 4D and Supplementary Fig. S6E). Thus, the 60-kDa bands are likely the result of interaction of C46 via a disulfide bond with another HokB monomer or other proteins.

Finally, the most striking result was observed when edited HokB was expressed in the presence of the C46S substitution (Fig. 4D and Supplementary Fig. S6E). We observed multiple strong bands above the expected molecular weight of monomeric HokB, which disappeared in the presence of DTT. Moreover, these bands were weaker to not visible when the HokB (Y29#) and non-edited HokB (Y29) were expressed, with C46 or with S46. Thus, these bands are likely the product of disulfide bonds between edited HokB and other proteins via C29, but only when C46 is not present. In other words, C29 and C46 are bound via disulfide bond, and when C46 is mutated to S46, C29 is free to interact with other cysteine residues in other proteins, as evident by the strong bands that are not visible upon treatment with DTT. Notably, the interaction has to be via C29, because these bands are lacking or barely visible when expressing the non-edited HokB (Y29) with S46 (Fig. 4D and Supplementary Fig. S6E).

To conclude, our data support the notion that A-to-I mRNA editing in HokB mediates intramolecular disulfide bond formation between C29 (introduced by editing) and C46 (encoded at the DNA level). However, further work is needed to exclude that C29 interacts via disulfide bond with other cysteine residues of HokB large oligomers or other proteins that are not captured by our analysis.

Also shown are samples without mCherry or HokB induction (Ara−). When induced (Ara+), samples were prepared under reducing (DTT+) or non-reducing (DTT−) conditions. Notice the lack of visible bands that match the size of mCherry (27 kDa) when induced and not induced, as mCherry is a cytoplasmic protein. Outer membrane protein A (OmpA; ≈27 kDa) was used as a membrane marker, supporting that we analyzed the membrane protein fraction (together with the lack of signal from *E. coli* expressing mCherry alone). Orange arrows mark the band with the expected mass of mCherry-HokB. Black arrows mark the bands in the size of 55–60 kDa that may represent a truncated HokB dimer that is sensitive to reducing conditions (DTT+). Purple arrows mark bands above the expected molecular weight of monomeric HokB sensitive to reducing conditions (DTT+) observed when edited HokB was expressed in the presence of the C46S substitution.

### High levels of edited HokB induce bacterial death

**Figure 5.**
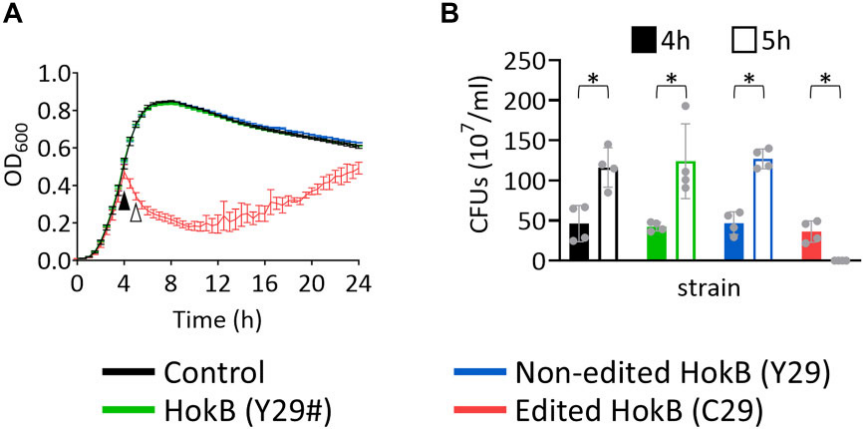
High levels of edited HokB induce bacterial death. (**A**) Strains were allowed to grow without induction for 4 h. Then, arabinose was added to induce HokB protein expression (the black arrow marks the induction time). Presented are OD values over time. The expression of mCherry and HokB was induced with 0.2% arabinose from a pBAD vector. The mean and standard errors of three biological replicates conducted on different days (*N* = 3), each with 21 technical replicates, are shown. Black and white triangles correspond to sampling times for panel (B). (**B**) CFU (colony forming unit) counts before and after 1 h of induction (CFUs at 4 and 5 h). Only 0.3% of viable bacteria survived after 1 h of edited HokB induction (corresponding to the death of 99.7% of viable bacteria). The mean and standard error of four biological replicates conducted on different days (*N* = 4) are shown. Statistical analysis was conducted using Student’s paired *t*-test followed by Benjamini–Hochberg FDR correction: *P*-value ≤.05 (*).

The phenotype induced by edited HokB (Figs 1 and 2) can be caused by growth inhibition or bacterial death. To test these two alternatives, we grew bacteria for 4 h without plasmid-borne HokB expression and then induced the expression of the different versions of HokB. We observed no toxic effect in the strains expressing HokB or non-edited HokB (Fig. 5A and Supplementary Fig. S7A). In contrast, we observed a decline in optical density (OD_600_) in the strain expressing edited HokB (Fig. 5A and Supplementary Fig. S7), indicating that edited HokB toxicity is mediated by a killing effect and cell lysis rather than growth arrest. Similarly, we observed the same phenotype (i.e. a decrease in OD levels) when we coexpressed TadA with the editable variant of *hokB* (Supplementary Fig. S7B). To validate that bacteria are truly dying, we also counted viable bacterial cells (CFUs) 1 h after induction (after a total of 5 h). The strain expressing the control, HokB, and non-edited HokB displayed an increase in CFUs 1 h after induction (Fig. 5B). In contrast, we observed a near-complete loss of viable bacterial cells in the strain expressing edited HokB, as only 0.3% of CFUs remained after 1 h of induction, corresponding to the death of 99.7% of viable bacteria observed after 4 h of growth (Fig. 5B). We conclude that edited HokB induces bacterial cell death when highly expressed.

### Lower levels of edited HokB induce early entrance to the stationary phase

What is the effect of lower levels of edited HokB expression? Until this point, we examined the effect of highly expressing the different versions of HokB. This experimental design (0.2% arabinose) increased the RNA expression (measured by qRT-PCR) encoding HokB and non-edited HokB by three orders of magnitude on average, but the RNA levels encoding edited HokB increased by only two orders of magnitude (6394-, 4038-, and 418-fold, respectively; Supplementary Fig. S8). We conclude that edited HokB can induce cell death and that such a killing effect can be achieved by only 1/10 of the RNA expression level of edited *hokB* as compared to the RNA expression of the non-edited version (Supplementary Fig. S8).

**Figure 6.**
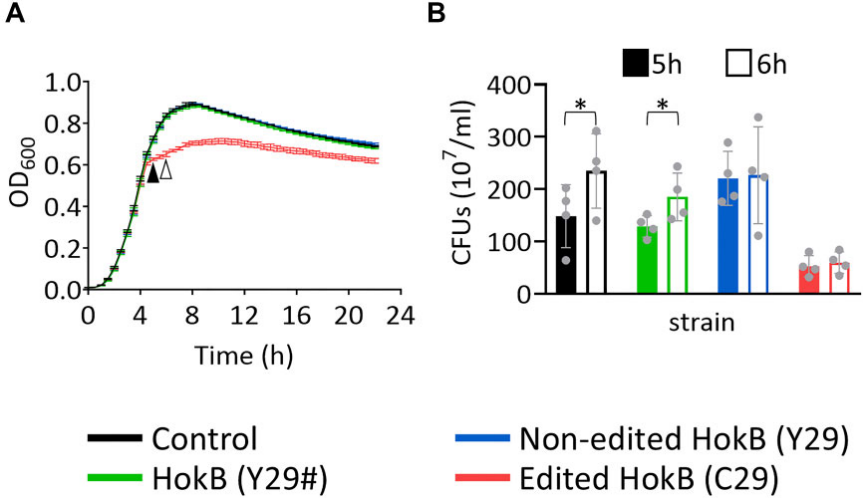
Lower levels of edited HokB induce early entrance to the stationary phase. (**A**) Growth analysis of WT *E. coli* as described in Fig. 2C with 1:1000 lower arabinose concentration. The expression of mCherry and HokB was induced from the beginning of the experiment (time point “0”) with 0.0002% arabinose from a pBAD vector. Black and white triangles correspond to sampling times for panel (B). (**B**) CFU counts at 5 and 6 h of the beginning of growth. Notice that there are fewer CFUs when edited HokB is expressed, with similar numbers at 5 and 6 h after growth. The mean and standard error of four biological replicates conducted on different days (*N* = 4) are shown. Statistical analysis was conducted using Student’s paired *t*-test followed by Benjamini–Hochberg FDR correction: *P*-value ≤.05 (*).

To examine the effect of edited HokB in lower levels of expression, which are likely to be more relevant physiologically, we induced its expression with 1:1000 lower arabinose concentration (0.0002%; the lowest concentration shown to elicit a phenotype when using the pBAD-mCherry-HokB vector [42]). We observed that at this low arabinose concentration, the mean expression of the RNA encoding HokB and non-edited HokB increased by two orders of magnitude compared to the endogenous expression level of *hokB* in the control strain (240- and 289-fold, respectively; Supplementary Fig. S8). In contrast, the mean expression of the RNA encoding edited HokB increased by only one order of magnitude (64-fold; Supplementary Fig. S8), which may represent a more realistic level of endogenous *hokB* expression under certain physiological conditions. We observed no toxic effect at the lower expression level when expressing HokB and non-edited HokB, as they display similar growth kinetics as the control (Fig. 6A and Supplementary Fig. S9A). However, expressing edited HokB induced early entrance to the stationary phase (Fig. 6A and Supplementary Fig. S9A). Furthermore, CFU numbers did not significantly change between 5 and 6 h following edited HokB expression, supporting early entrance to the stationary phase (Fig. 6B). Moreover, edited HokB did not induce early entrance to the stationary phase in the Δ*dsbA* strain (Supplementary Fig. S9B). Finally, we also observed overexpressing TadA together with the editable variant of *hokB* in lower induction levels induced early entrance to the stationary phase (Supplementary Fig. S9C and D). Early entrance to the stationary phase was observed with slightly higher arabinose concentration (0.002% instead of 0.0002%), compared to expressing the edited version of HokB without expressing TadA, which we interpret as the result of the different experimental setup (two-plasmid system instead of one, and that the product of one plasmid, edited HokB, is dependent on the product of another plasmid, TadA). To conclude, disulfide bond formation activity is required for enhanced toxicity of edited HokB in lower induction levels, manifested by the early entrance of the culture into the stationary phase.

### A-to-I mRNA editing of *hokB* is conserved in pathogenic *E. coli* and *Shigella* strains

**Figure 7.**
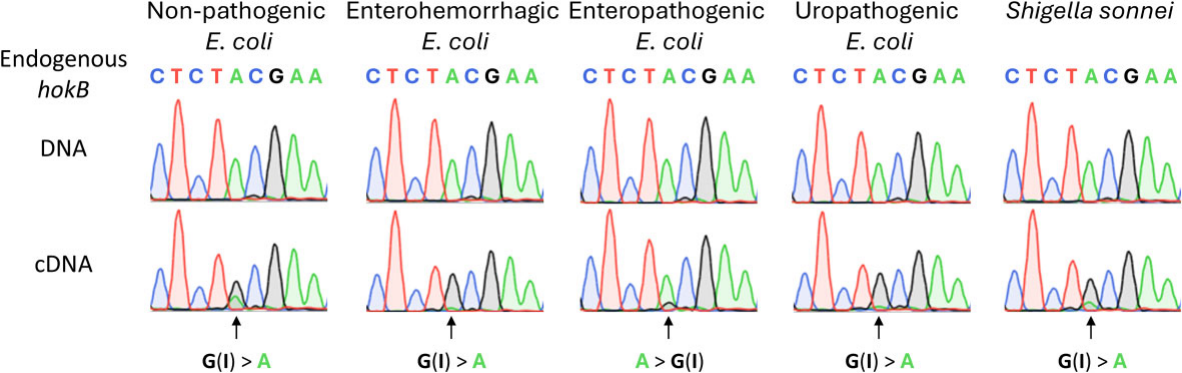
A-to-I mRNA editing of *hokB* is conserved in pathogenic *E. coli* and *Shigella* strains. Sanger sequencing of the endogenous *hokB* gene and its mRNA from the same sample of non-pathogenic *E. coli* (used throughout this work), enterohemorrhagic *E. coli*, enteropathogenic *E. coli*, uropathogenic *E. coli*, and *Shigella sonnei*. A black arrow marks the double peak of A and G(I) observed only in the cDNA samples. Note that the G(I) peak (black) is higher than the A peak (green) in most samples. Sequences were aligned to the *E. coli* reference genome (NC_000913.3) and positions 1491982–1491990 are shown. See Supplementary Fig. S10 for exact genomic coordinates of the full-length *hokB* gene in each species.

Next, we aimed to examine whether A-to-I mRNA editing of *hokB* is conserved in other strains and species. We focused on five strains: non-pathogenic *E. coli* (used throughout this work as a reference strain), enterohemorrhagic *E. coli*, enteropathogenic *E. coli*, uropathogenic *E. coli*, and *Shigella sonnei*. All strains encode *hokB* in their genome with a tyrosine codon (TAC) at the homologous position for tyrosine/cysteine 29 focused in this work (Fig. 7). By sequencing DNA and RNA (cDNA) from the same sample, we identified that A-to-I mRNA editing of *hokB* occurs in all tested species (Fig. 7). Moreover, most strains (except for enteropathogenic *E. coli*) harbored high levels of editing in *hokB*, recoding a tyrosine to a cysteine codon at position 29 of HokB. Interestingly, enteropathogenic *E. coli* harbors a five-base deletion in the coding sequence of *hokB* (positions 1492060–1492064 in NC_000913.3; Supplementary Fig. S10A). This deletion changes the protein sequence of HokB, starting from the fourth amino acid and results in a premature stop codon (Supplementary Fig. S10B). Thus, enteropathogenic *E. coli* probably does not encode a functional HokB protein. Subsequently, the lack of encoded HokB suggests that editing (in *hokB*) does not serve a function in enteropathogenic *E. coli*, providing a possible hint to the observed lower editing levels (Fig. 7). Notably, the deletion does not affect the predicted secondary structure of *hokB* around the edited site (Supplementary Fig. S10C). Thus, another molecular mechanism accounts for the lower editing levels in enteropathogenic *E. coli*, possibly TadA expression.

In conclusion, A-to-I mRNA editing of *hokB* is conserved in pathogenic species and strains, supporting functional importance with possible clinical relevance.

The expression of mCherry and HokB was induced from the beginning of the experiment (time point “0”) with 0.2% arabinose from a pBAD vector. Expression of DsbA was induced with 1 mM IPTG from a pME6032 vector. The mean and standard errors of three biological replicates conducted on different days (*N* = 3), each with 21 technical replicates, are shown.

## Discussion

A-to-I mRNA editing was shown to play an essential role in various cellular processes and diseases in eukaryotes [1, 3, 5–10, 12–30]. Here, we show experimentally for the first time that A-to-I mRNA editing in bacteria can directly affect protein sequence, function, and disulfide bond formation. Furthermore, we show that A-to-I mRNA editing can potentially affect bacterial growth and viability and is conserved in strains and species with clinical relevance.

To the best of our knowledge, our work reveals a novel and unexplored function of A-to-I mRNA editing in regulating disulfide bond formation. However, what is the purpose of the editing-controlled disulfide bond in HokB? A clue to this question might reside in the inability of DsbC expression to rescue the growth of *E. coli* expressing edited HokB (Supplementary Fig. S5). This is unexpected as DsbC was shown to break disulfide bonds between monomers of the non-edited version of HokB (between cysteines at position 46) [44]. Thus, the disulfide bond in edited HokB may protect against DsbC activity. Furthermore, our western blot analysis supports the notion that the editing-controlled disulfide bond is intramolecular, i.e. within HokB monomers, between positions C29 and 46 (Fig. 4). Thus, it is possible that this disulfide bond may be necessary to maintain the folding of the monomer in the periplasm part of HokB. In turn, the disulfide bond may be necessary to maintain a conformation required for pore formation.

### However, why control disulfide bond formation at the RNA level and not hardcode it into the genome?

Previously, we discovered that mRNA editing levels in *hokB* increase as bacterial density increases within the culture [37]. Furthermore, *hokB* expression is controlled by the presence of the ppGpp, a molecule formed when bacteria experience starvation [42]. Combined, mRNA editing may provide a handle that regulates the toxicity level of HokB at unknown physiological conditions via disulfide bond formation. In other words, mRNA editing may enable bacteria to tune not only the expression of HokB but also its toxicity.

Here, we used the plasmid-borne system to demonstrate the potential role of editing in bacteria. However, we observe that only 13%–27% of the plasmid-borne transcripts of *hokB* are edited (Fig. 1D and Supplementary Fig. S3A). In contrast, when *hokB* is endogenously expressed from the chromosome, up to 90% of the transcripts are edited [37]. What can account for this difference in editing level? One reason is that the expression levels of endogenous TadA are not sufficient to edit the overexpressed plasmid-borne transcripts. Indeed, when we supplement TadA from a second plasmid, we rescue the editing level of plasmid-borne *hokB* to be similar to endogenous editing level (Fig. 1D–E). Alternatively, a recent study has shown that RNA stability is an important feature for editing in *S. pyogenes* [38]. Thus, it could be that the plasmid-borne mRNA of *hokB* that lacks its 5′-region is less stable than its chromosome counterpart. Subsequently, when expressed from a plasmid, *hokB* is edited to a lower extent (Fig. 1D and E).

The translation of HokB is constitutively blocked in most bacterial cells in the population by its cognate antitoxin *sokB* that binds to the 5′-untranslated region and prevents the translation of HokB [42]. That is the reason the plasmid system we used includes only the coding sequence for HokB without the 5′-untranslated region, enabling us to express and control the levels of HokB. Furthermore, the mCherry tag helped us to validate the expression of the different HokB versions in the different strains we used. For example, because we used mCherry fused to HokB, we could observe that the expression of edited HokB was rescued in the Δ*dsbA* mutant *in vivo*. This result supports the notion that the toxic effect is because of DsbA activity and not because of an unknown effect on the expression of edited HokB. Importantly, the mild toxicity observed by expressing mCherry-HokB without co-expressing TadA is similar to the recently observed phenotype when expressing HokB with a shorter tag (His) [56]. Moreover, HokB pore and persister formation were similar when mCherry or His-tag were fused to HokB [42, 44]. Thus, the mCherry tag likely does not interfere with HokB’s activity. Nevertheless, future work must examine the effect of editing within *hokB* in its natural context when expressed from the chromosome. To this end, one should identify conditions that elicit a phenotype in the presence and absence of *hokB* in the genome and the presence and absence of editing. Only then could the biological significance of editing within *hokB* be precisely examined.

Finally, A-to-I mRNA editing was reported to occur in *E. coli*, *Klebsiella pneumoniae*, and *S. pyogenes* [37, 38, 40]. Furthermore, some editing events are conserved between different strains of the same species [38, 40]. Previously, we detected A-to-G mismatches at the transcripts encoding Hok-family homologs in RNA-seq datasets of *Yersinia enterocolitica* and *K. pneumoniae* [37]. However, we did not have corresponding DNA samples to truly identify them as *bona fide* A-to-I mRNA editing events. Here, by using corresponding DNA and RNA samples, we observed that editing of *hokB* is conserved in *E. coli* and *Shigella* strains (Fig. 7). In turn, this observed conservation of editing could support functional importance in *E. coli* and *Shigella* strains with possible clinical relevance. Future work should examine editing occurrence and conservation across multiple species as the first step to understanding the role of A-to-I mRNA editing in bacteria.

In conclusion, mRNA editing research in bacteria is a relatively new field. Our work implicates mRNA editing as a new mechanism to affect protein sequence, function, and disulfide bond formation in bacteria, possibly impacting bacterial growth, viability, and pathogenicity.

## Supplementary Material

gkaf584_Supplemental_Files

## Data Availability

Data are available in a public, open-access repository. The data were deposited to the Pride database through the ProteomeXchange in project accession: PXD051162. The amplicon sequencing data were deposited to the NCBI SRA under accession PRJNA1227425.

## Supplementary data

Supplementary data is available at NAR online.
