# Supplementary material for: A-to-I mRNA editing in bacteria can affect protein sequence, disulfide bond formation, and function
Source: Nucleic Acids Res. 2025 Jul 2;53(12):gkaf584. doi: 10.1093/nar/gkaf584 (PMC12214031; doi:10.1093/nar/gkaf584)
Supplement: gkaf584_Supplemental_Files [file gkaf584_supplemental_files.zip › Didi et al SuppText and Figures_NAR_Revised_2.pdf]

A-to-I mRNA editing in bacteria can affect protein sequence, disulfide bond formation, and function

Liron Didi[1], Ofir Fargeon[1], Liam Aspit[1], Eyal Elias[1], Dor Braverman[1], Dganit Melamed[2], Daniel Keidar-Friedman[3], Nadav Sorek[3], Orit Raz[3], Sharon Ovnat Tamir[3], Raz Zarivach[4], Neta Sal-Man[1], Orna Dahan[5], Yitzhak Pilpel[5*], and Dan Bar Yaacov[1*]

[1]The Shraga Segal Department of Microbiology, Immunology and Genetics, Faculty of Health Sciences, Ben-Gurion University of the Negev, Beer-Sheva, Israel; [2]The Smoler Protein Research Center, Technion Israel Institute of Technology, Israel; [3]Assuta Ashdod University Hospital, Faculty of Health Sciences, Ben Gurion University of the Negev; [4]Department of Life Sciences, Ben-Gurion University of the Negev, Israel; [5]Department of Molecular Genetics, Weizmann Institute of Science, Rehovot, Israel
*Correspondence: pilpel@weizmann.ac.il and danbary@bgu.ac.il

SUPPLEMENTARY FIGURES AND TABLES' legend

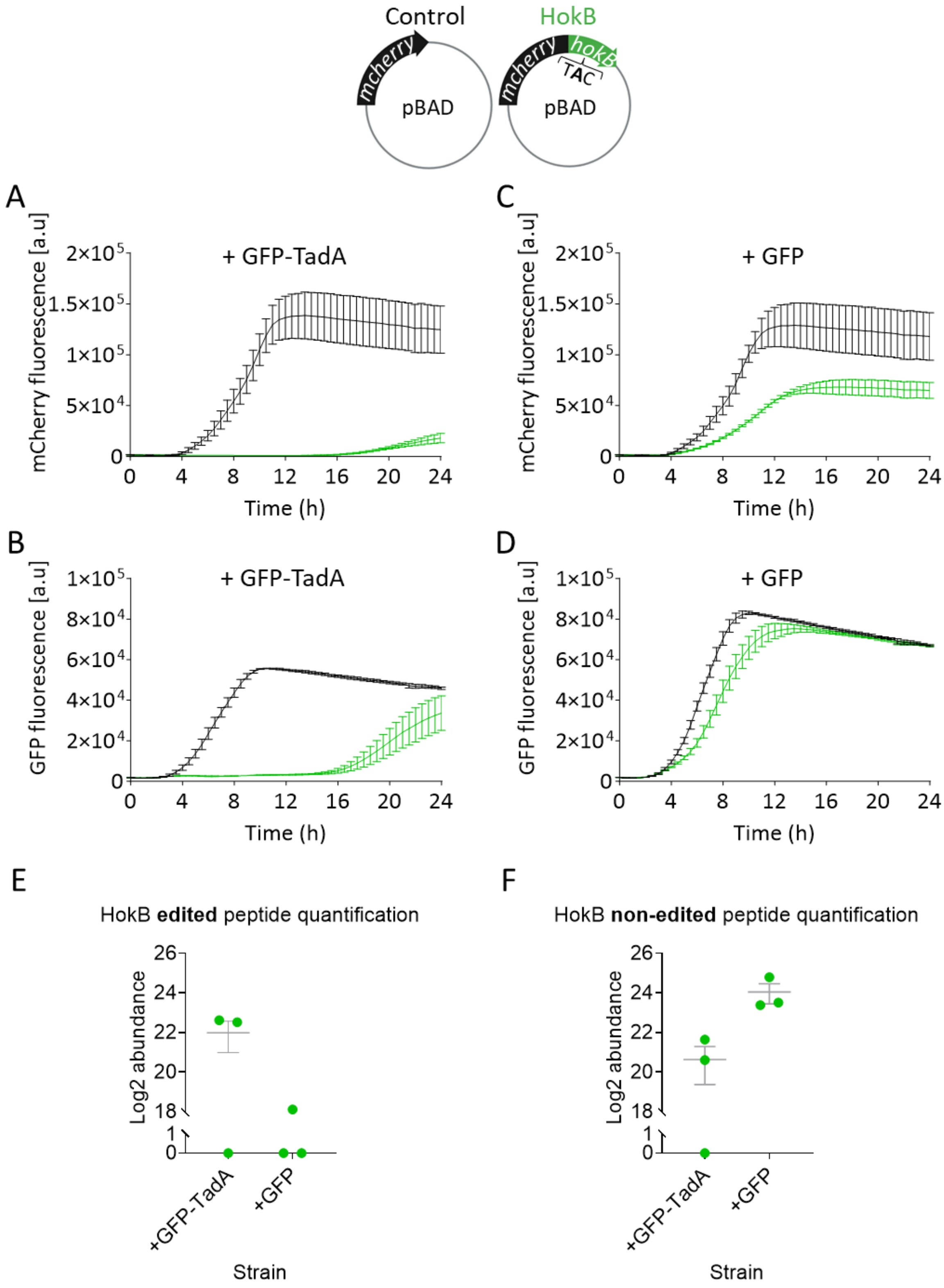
Control
HokB
mcherry
hokB
TAC
pBAD
A
+ GFP-TadA
mCherry fluorescence [a.u]
Time (h)
C
+ GFP
B
GFP fluorescence [a.u]
D
E
HokB **edited** peptide quantification
Log2 abundance
+GFP-TadA
+GFP
Strain
F
HokB **non-edited** peptide quantification

Supplementary Figure 1. Validation of HokB expression by mCherry and GFP fluorescence of Figure 1C. **A.** Levels of mCherry in the strains co-overexpressing mCherry (black) or mCherry-HokB (green) with GFP-TadA. The levels of mCherry start to rise after 16 hours likely because no growth was observed up to this time (Figure 1C). **B.** Levels of GFP in the strains co-overexpressing mCherry (black) or mCherry-HokB (green) with GFP-TadA. The levels of GFP start to rise after 16 hours likely because no growth was observed up to this time (Figure 1C). **C.** Levels of mCherry in the strains co-overexpressing mCherry (black) or mCherry-HokB (green) with GFP. **D.** Levels of GFP in the strains co-overexpressing mCherry (black) or mCherry-HokB (green) with GFP. For A-D the mean and standard error of three biological replicates conducted on different days (N = 3), each with 21 technical replicates are shown. Fluorescence levels are normalized to the average of 12 blank wells on the plates (medium only, no bacteria added). Thus, a value of "0" represents similar fluorescence levels as the blank wells. **E.** Mass spectrometry quantification of the edited peptide upon co-overexpression of HokB and GFP-TadA or GFP. In Two samples co-overexpressing HokB and GFP and in one sample co-overexpressing HokB and GFP-TadA we did not observe the edited peptide. **F.** Mass spectrometry quantification of the non-edited peptide upon co-overexpression of HokB and GFP-TadA or GFP. In one of the samples co-overexpressing HokB and GFP-TadA we did not observe the non-edited peptide. Throughout this Figure the expression of mCherry and HokB was induced with 0.2% arabinose from a pBAD vector. Expression of GFP-TadA or GFP was induced with 1mM IPTG from a pME6032 vector.

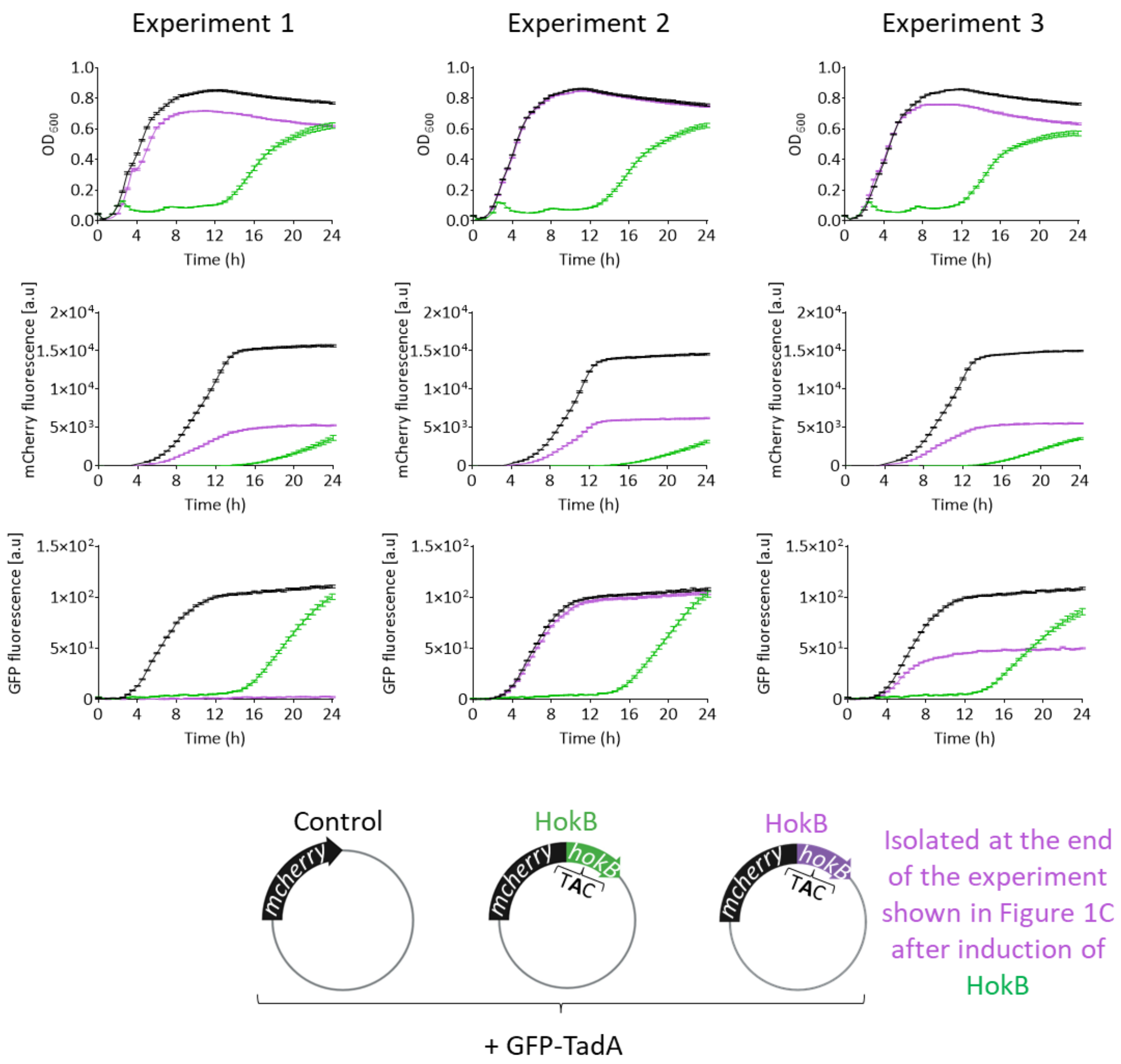

Supplementary Figure 2. The delayed growth observed upon edited HokB expression is mediated by bacteria harboring genetic mutations. Growth, mCherry, and GFP expression analysis of *E. coli* (Top10-DH10B) co-expressing mCherry (control-black) or mCherry-HokB (green and purple), with GFP-TadA. The purple line represents measurements of strains isolated at the end of the growth experiment shown in Figure 1C (left panel), which is similar to the delayed growth shown here in green (as control to make sure that growth in the purple line is not due to problems in the experiment). Each growth assay is conducted with bacteria from different colonies on the plate. Notice that in all three replicates mCherry is observed at the mutant strains (purple), but not in the naïve strain that is expressing HokB for the first time. Notice that in experiment 1 and 3 there is no or lower GFP signal, respectively, suggesting that TadA-GFP expression is shut off or reduced, probably affecting editing in *hokB* and subsequent toxicity. In experiment 2 GFP-TadA is expressed, suggesting a different solution to prevent the toxicity of HokB.

The mean and standard errors of 21 technical replicates, per experiment, are shown. The expression of mCherry and HokB was induced from the beginning of the experiment (time point “0”) with 0.2% arabinose from a pBAD vector. Expression of GFP-TadA was induced with 1 mM IPTG from a pME6032 vector. Each experiment (replicate) was done on different isolated colonies at different days.

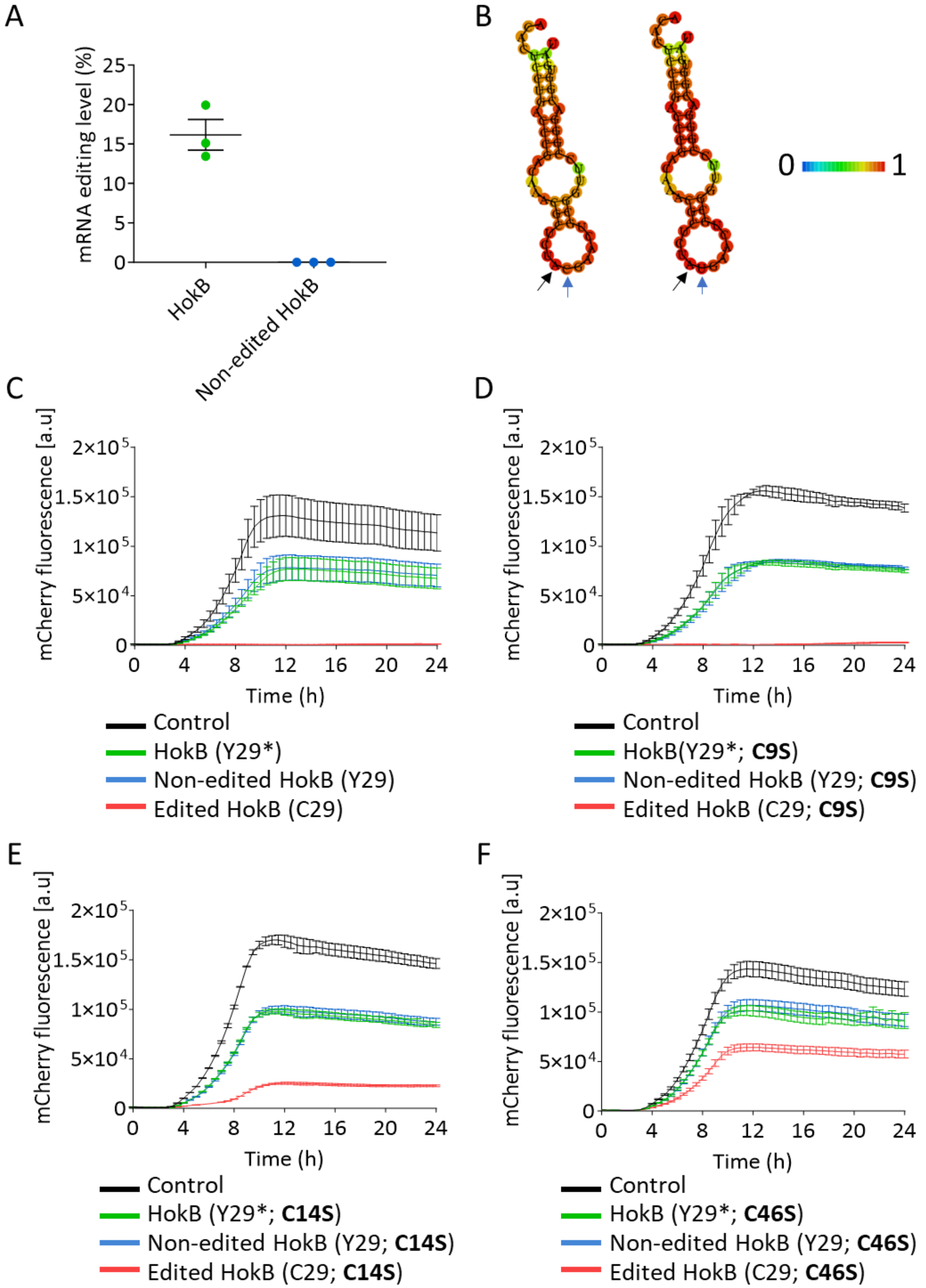
A
mRNA editing level (%)
25
20
15
10
5
0
HokB
Non-edited HokB
B
0
1
C
mCherry fluorescence [a.u]
2×10^5
1.5×10^5
1×10^5
5×10^4
0
0 4 8 12 16 20 24
Time (h)
Control
HokB (Y29*)
Non-edited HokB (Y29)
Edited HokB (C29)
D
mCherry fluorescence [a.u]
Time (h)
Control
HokB(Y29*; C9S)
Non-edited HokB (Y29; C9S)
Edited HokB (C29; C9S)
E
mCherry fluorescence [a.u]
Time (h)
Control
HokB (Y29*; C14S)
Non-edited HokB (Y29; C14S)
Edited HokB (C29; C14S)
F
mCherry fluorescence [a.u]
Time (h)
Control
HokB (Y29*; C46S)
Non-edited HokB (Y29; C46S)
Edited HokB (C29; C46S)

Supplementary Figure 3. Validation of plasmid-born *hokB* editing levels and HokB expression by mCherry fluorescence of Figure 2. A. Editing levels of plasmid-born *hokB* transcript as determined by Amplicon-sequencing. HokB (Y29#) is in green and non-edited HokB (Y29) is in blue. A detection threshold of 0.01% was used as the minimum value to report editing. Minimum observed reads coverage per sample that passed our quality filters ≥ 300,000. RNA was purified after 3.5 hours of growth as performed in Figure 2C. Shown are the average and standard errors for three biological replicates (conducted on different days), per experiment. **B.** The non-edited transcript of *hokB* having a TAT codon for the amino acid at position 29 (Y29) is predicted to form a similar stem-loop structure as the WT version harboring the original TAC codon (left to right, respectively). The black arrow marks the position of the edited adenosine and the blue arrow the mutated position. Prediction was made using 25 upstream and downstream bases of the edited adenosine as we did before using RNAfold (37, 58). The color-code shows base-pair probabilities as calculated by RNAfold (58). **C.** mCherry signal from the growth analysis performed in Figure 2C. Note that mCherry signal is observed from all strains except for the strain expressing edited HokB. We previously showed that this is due to the enhanced toxicity effect of edited HokB, that prevent sufficient accumulation of mCherry-HokB C29 protein to be detected by the plate reader (37). **D.** Same as in B but for variants containing cysteine to serin substitution at position 9 (refers to Figure 2D). **E.** Same as in B but for variants containing cysteine to serin substitution at position 14 (refers to Figure 2E). Note that mCherry signal is now observed from all strains, including the strain expressing edited HokB. **F.** Same as in B but for variants containing cysteine to serin substitution at position 46 (refers to Figure 2F). Note that mCherry signal is now observed from all strains, including the strain expressing edited HokB. Error bars represent standard error of three biological replicates conducted in different days (N = 3), each with 21 technical replicates (except in the case of C14S, where fluorescence measurements were taken from two biological replicates). Fluorescence levels are normalized to the average of 12 blank wells on the plates (medium only, no bacteria added). Thus, a value of "0" represents similar fluorescence levels as the blank wells.

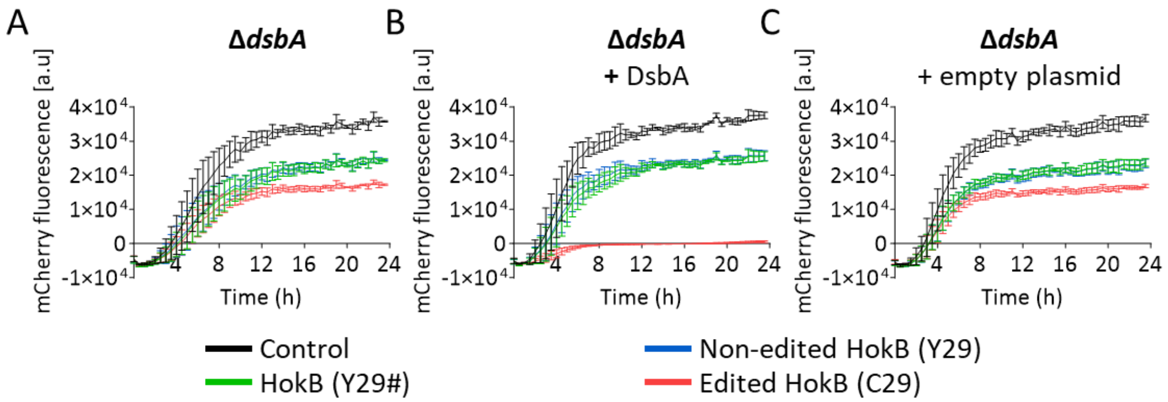

Supplementary Figure 4. Validation of HokB expression by mCherry fluorescence of Figure 3. A. mCherry signal from the growth analysis performed in Figure 3A. Note that mCherry signal is observed from all strains, including the strain expressing edited HokB, unlike in Supplementary Figure 1A. **B.** Same as in A but with overexpressing DsbA from a second plasmid (pME6032; refers to Figure 3B). **C.** Growth analysis, as in B, using an empty plasmid (pME6032 with no *dsbA* insert; (refers to Figure 3C).

All growth experiments were conducted as explained in Figure 2. Fluorescence levels are normalized to the average of 12 blank wells on the plates (medium only, no bacteria added). Thus, a value of "0" represents similar fluorescence levels as the blank wells. Notably, from unknown reasons the fluorescence at the beginning of growth was lower in bacteria-containing wells than in the blank wells, resulting in negative values at the start of the experiments. Nevertheless, it still validates the expression of HokB fused to mCherry as fluorescence rise as the experiments continues (except for edited HokB in panel B, which is not visible due to the toxicity of edited HokB that hinders proper growth and accumulation of fluorescent signal).

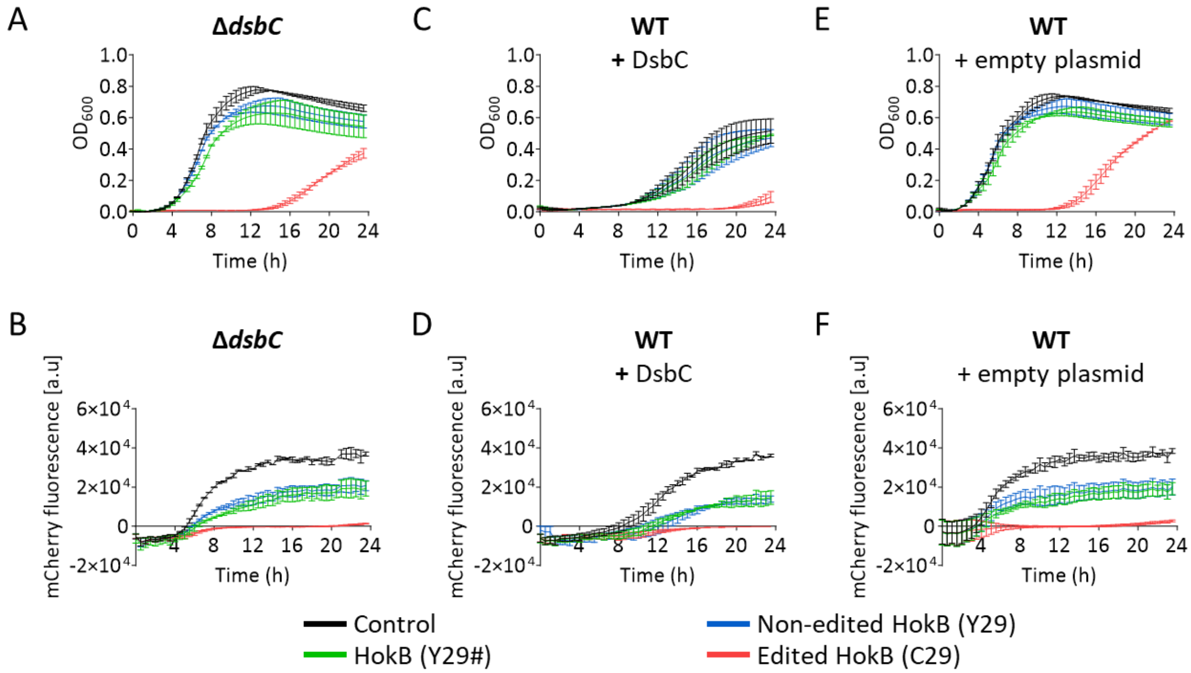

Supplementary Figure 5. DsbC activity does not affect the toxicity of the edited HokB. **A.** Growth analysis of an *E. coli* Δ*dsbC* strain that expresses one of three versions of HokB, fused to the mCherry reporter protein from an inducible plasmid. As a reference control, we used a plasmid encoding only mCherry. **B.** mCherry signal from the growth analysis performed in A. **C.** Growth analysis as in A, but in the WT strain, overexpressing DsbC from a second plasmid (pME6032). **D.** mCherry signal from the growth analysis performed in C. **E.** Growth analysis as in C, using an empty plasmid (pME6032 with no *dsbC* insert). **F.** mCherry signal from the growth analysis performed in E.

The expression of mCherry and HokB was induced with 0.2% arabinose from a pBAD vector. Expression of DsbC was induced with 1mM IPTG from a pME6032 vector. Error bars represent standard error of two biological replicates conducted in different days (N = 2), each with 21 technical replicates.

Fluorescence levels are normalized to the average of 12 blank wells on the plates (medium only, no bacteria added). Thus, a value of "0" represents similar fluorescence levels as the blank wells. Notably, as in Supplementary Figure 4, for unknown reasons the fluorescence at the beginning of growth was lower in bacteria containing wells than in the blank wells, resulting in negative values at the start of the experiments. Nevertheless, it still validates the expression of HokB fused to mCherry as fluorescence rise as the experiments continues (except for edited HokB, which is not visible due to the toxicity of edited HokB that hinders proper growth and accumulation of fluorescent signal).

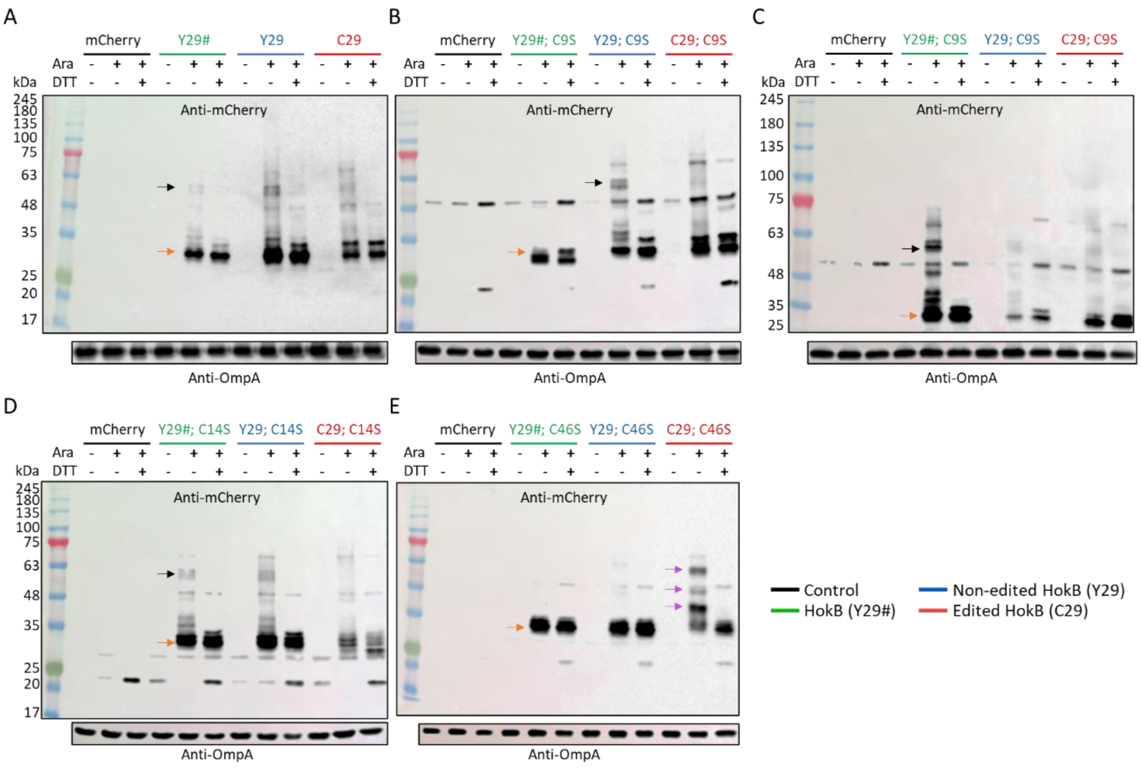

Supplementary Figure 6. A second repeat of the western blot analysis supports that A-to-I mRNA editing mediates an intramolecular disulfide bond between C29 and C46 in HokB. A. Western blot of membrane enriched protein fraction of *E. coli* (Top10-DH10B) WT strain expressing either mCherry (control; black) or the HokB (Y29#, green), non-edited HokB (Y29, blue), and edited HokB (C29, red) fused to mCherry reporter protein (N-terminus) from the plasmid shown in Figure 2B. **B-C**. Same as A but with the C9S substitution in the different expressed HokB versions. Notably, the Y29#; C9S expressing strain in the second repeat (shown in B) does not behave similarly as the first repeat (Figure 4B). Therefore, we repeated this experiment a third time (shown in C; note that the gel run for a longer period of time). In this repeat, the Y29#; C9S expressing strain is similar to what observed in the first repeat (Figure 4B). However, the bands in the Y29; C9S and C29; C9S expressing strains are fainter. We interpret this as the likely result of technical issues during sample preparation, because we had to induce HokB expression for only 90 seconds (otherwise the C29 expressing strains, in A and B, will undergo lysis due to the toxicity of C29 containing HokB). **D**. Same as A but with the C14S substitution in the different expressed HokB versions. **E**. Same as A but with the C46S substitution in the different expressed HokB versions.

Also shown are samples without mCherry or HokB induction (Ara -). When induced (Ara +), samples were prepared under reducing (DTT +) or non-reducing (DTT -) conditions. Notice the lack of visible bands that match the size of mCherry (27 kDa) when induced and not induced, as mCherry is a cytoplasmic protein. Outer membrane protein A (OmpA) was used as a membrane marker supporting that we analyzed the membrane protein fraction (together with the lack of signal from *E. coli* expressing mCherry alone). Orange arrows mark the band with the expected mass of mCherry-HokB. Black arrows mark the bands in the size of 55-60 kDa that may represent a truncated HokB dimer that is sensitive to reducing conditions (DTT +). Purple arrows mark bands above the expected molecular weight of monomeric HokB that are sensitive to reducing conditions (DTT) observed when edited HokB was expressed in the presence of the C46S substitution.

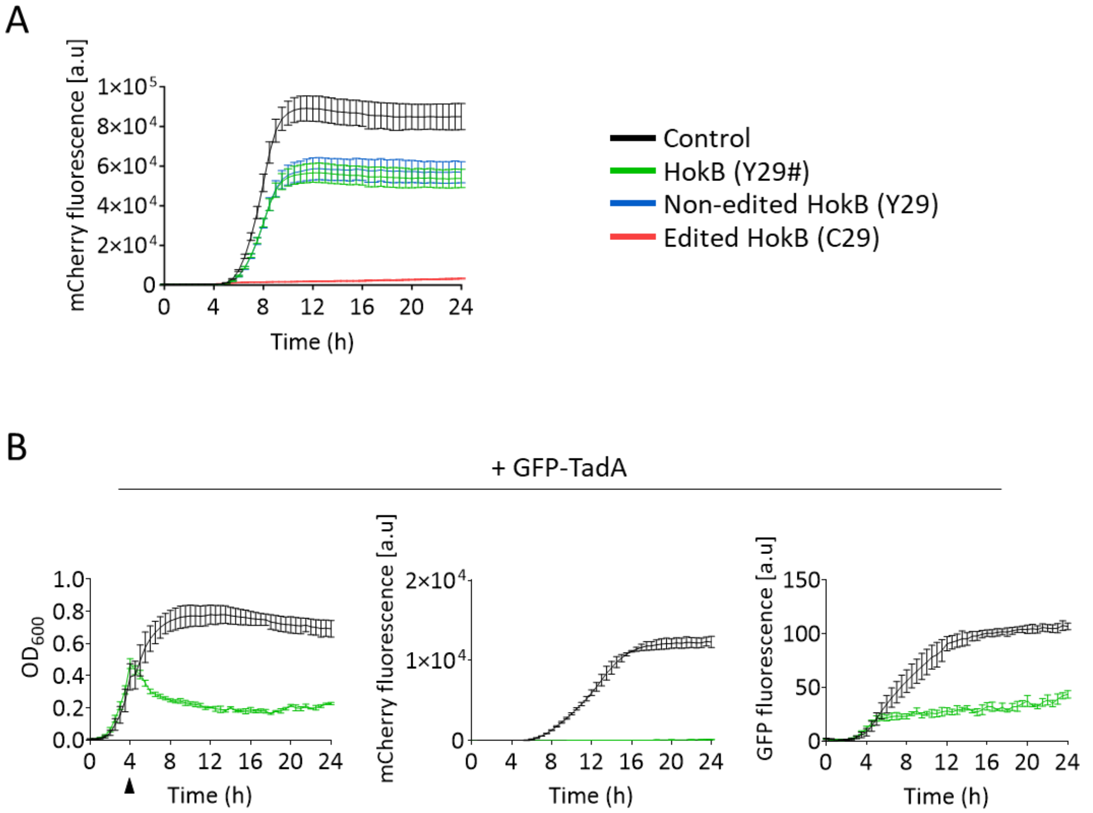

Supplementary Figure 7. High levels of edited HokB induce bacterial death. A. Validation of HokB expression by mCherry fluorescence of Figure 5A. The growth experiment was conducted as explained in Figure 5A and mCherry levels were detected over time. Error bars represent standard errors of three biological replicates conducted in different days (N = 3), each with 21 technical replicates. **B.** Growth and protein expression analysis of *E. coli* (Top10-DH10B) co-expressing mCherry (control-black) or mCherry-HokB (green), with GFP-TadA. The expression of mCherry and HokB was induced four hours after the beginning of the experiment (time point “4” marked with a black triangle) with 0.2% arabinose from a pBAD vector. The expression of GFP-TadA was induced with 1 mM IPTG from a pME6032 vector from the beginning of the experiment (time point “0”). Left panel: growth measurements (OD600); middle panel: mCherry fluorescence measurements; right panel: GFP fluorescence measurements. The mean and standard errors of three biological replicates conducted on different days (N = 3), each with 21 technical replicates, are shown.

Fluorescence levels are normalized to the average of 12 blank wells on the plates (medium only, no bacteria added). Thus, a value of "0" represents similar fluorescence levels as the blank wells.

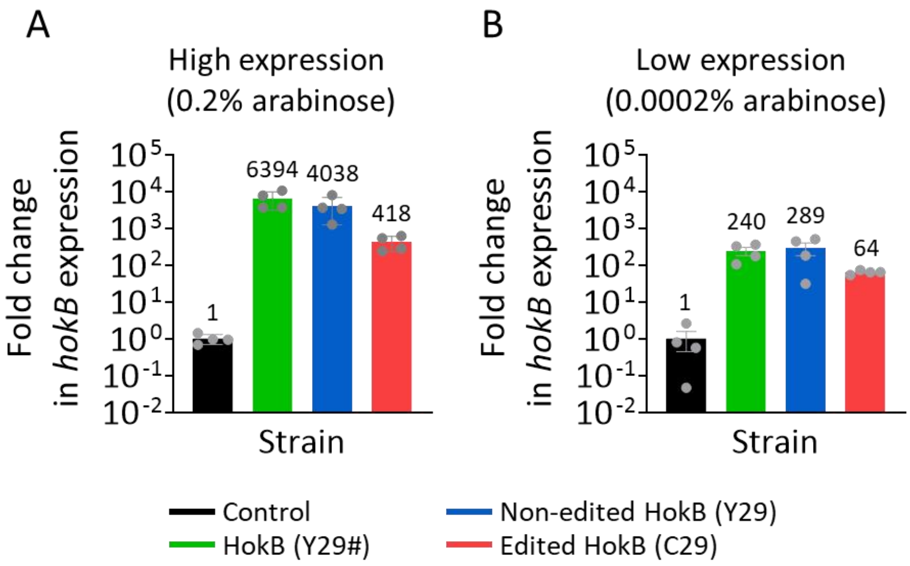

C

Protocol for Use with LightCycler® 480 Multiwell Plate 384

The following table shows the PCR parameters that must be programmed for a LightCycler® 480 System PCR run with the LightCycler® 480 SYBR Green I Master using a LightCycler® 480 Multiwell Plate 384:

Setup

Block Type	Reaction Volume [µl]
384	3-20

Detection Format	Excitation Filter	Emission Filter
SYBR Green / HRM Dye	465	510

Programs

Program Name	Cycles	Analysis Mode
Pre-Incubation	1	None
Amplification	45 [1)]	Quantification
Melting Curve	1	Melting Curve
Cooling	1	None

Temperature Targets

	Target [°C]	Acquisition Mode	Hold [hh:mm:ss]	Ramp Rate [°C/s]	Acquisitions [per °C]
Pre-Incubation	95	None	00:05:00 [2)]	4.8	–
Amplification	95	None	00:00:10	4.8	–
	primer dependent [3)]	None	00:00:05 - 00:00:20 [4)]	2.5 (Target °C ≥ 50°C)<br>2.0 (Target °C < 50°C) [7)]	–
	72	Single	00:00:05 - 00:00:30 [4) 5)]	4.8	–
Melting Curve	95	None	00:00:05	4.8	–
	65	None	00:01:00	2.5	–
	97	Continuous	–	–	5 - 10 [6)]
Cooling	40	None	00:00:10	2.0	–

Supplementary Figure 8. RT-qPCR of *hokB* in high and low arabinose concentration. Relative RNA levels of different *hokB* versions compared to the control strain (basal expression level) are presented on the Y axis. The normalizing gene (to which the expression of *hokB* is compared within each sample) is the transcript of RNA polymerase alpha subunit – *rpoA*). **A.** Growth was conducted in LB medium supplemented with 100 µg/ml ampicillin and 0.2% arabinose (final concentration). RNA was extracted after 2h and 15min. **B.** Growth was conducted in LB medium supplemented with 100 µg/ml ampicillin and 0.0002% arabinose (final concentration). RNA was extracted after 4h. Four biological replicates that were conducted on different days (N = 4) are shown. The numbers above each column represent the average of all four replicates per strain. **C.** protocol for use with LightCycler 480 Multiwell Plate 384.

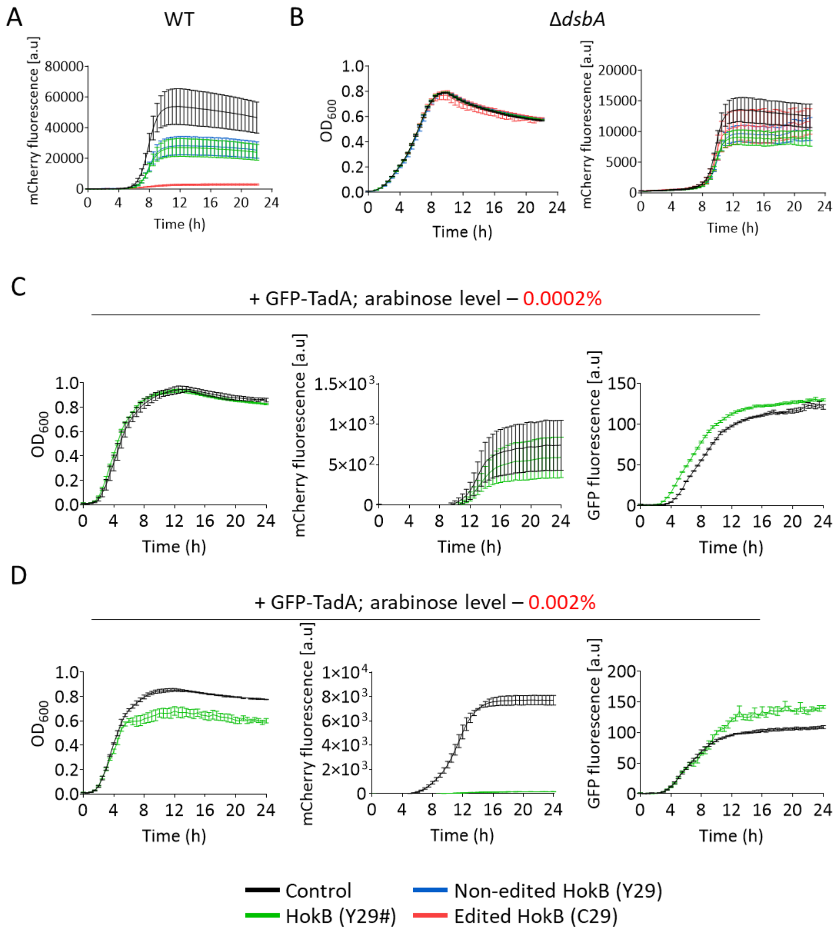

Supplementary Figure 9. Lower levels of edited HokB induce early entrance to the stationary phase in a DsbA-dependent manner. A. mCherry signal from the growth analysis performed in Figure 6A. The expression of mCherry and HokB was induced at the beginning of the experiment (time point "0" marked with a black triangle) with 0.0002% arabinose from a pBAD vector. **B.** Growth (left panel) and mCherry expression (right panel) analysis of *E. coli* (Top10-DH10B) as in Figure 6A and Supplementary Figure 9A, but in a Δ*dsbA* strain. The expression of mCherry and HokB was induced at the beginning of the experiment (time point "0" marked with a black triangle) with 0.0002% arabinose from a pBAD vector. **C**. Growth and protein expression analysis of *E. coli* (Top10-DH10B) co-expressing mCherry (control-black) or mCherry-HokB (green), with GFP-TadA. The expression of mCherry and HokB was induced at the beginning of the experiment (time point "0" marked with a black triangle) with 0.0002% arabinose from a pBAD vector. The expression of GFP-TadA was induced with 1 mM IPTG from a pME6032 vector from the beginning of the experiment (time point "0"). Left panel: growth measurements (OD600); middle panel: mCherry fluorescence measurements; right panel: GFP fluorescence measurements. **D**. Same as in C, but with arabinose concentration of 0.002%.

In all the panels shown are the average and standard errors of three biological replicates, conducted on different days (N = 3), each with 21 technical replicates. Fluorescence levels are normalized to the average of 12 blank wells on the plates (medium only, no bacteria added). Thus, a value of "0" represents similar fluorescence levels as the blank wells. The only exception was Supplementary Figure 9C in the GFP panel where we show the values of just one repeat, because we had a technical problem with our plate reader preventing it from detecting GFP efficiently.

A

NPEC 1 **ATG**AAGCACAACCCTCTGGTGGTGTGTCTGCTCATTATCTGCATTACGATTCTGACATTCACACTCCTGACCCGA 75
UPEC 1 **ATG**AAGCGCAACCCTCTGGTGGTGTGTCTGCTCATTATCTGCATTACGATTCTGACATTCACACTCCTGACCCGA 75
EHEC 1 **ATG**AAGCGCAACCCTCTGGTGGTGTGTCTGCTCATTATCTGCATTACGATTCTGACATTCACACTCCTGACCCGA 75
SHIG 1 **ATG**AAGCACAACCCTCTGGTAGTGTGTCTGCTCATTATCTGCATTACGATTCTGACATTCACACTCCTGACCCGA 75
EPEC 1 **ATG**AAGCGC-----TCTGGTGGTGTGTCTGCTCATTATCTGCATTACGATTCTGACATTCACACTCCTGACCCGA 70

NPEC 76 CAAACGCTCTACGAACTGCGGTTCCGGGACGGTGATAAGGAGGTTGCTGCGCTCATGGCCTGCACGTCCAGG**TAA** 150
UPEC 76 CAAACGCTCTACGAACTGCGGTTCCGGGACGGTGATAAGGAGGTTGCTGCGCTCATGGCCTGCACGTCCAGG**TAA** 150
EHEC 76 CAAACGCTCTACGAACTGCGGTTCCGGGACAGTGATAAGGAGGTTGCTGCGCTCATGGCCTGCACGTCCAGG**TAA** 150
SHIG 76 CAAACGCTCTACGAACTGCGGTTCCGGGACGGTGATAAGGAGGTTGCTGCGCTCATGGCCTGCACGTCCAGG**TAA** 150
EPEC 71 CAATCGCTCTACGAACTGCGGTTCCGGGACGG**TGATAA**GGAAGTTGCTGCGCTCATGGCCTGCACGTCCAGG**TAA** 145

B

NPEC 1 MKHNPLVVCLLIICITILTFTLLTRQTLYELRFRDGDKEVAALMACTSR 49
UPEC 1 MKRNPLVVCLLIICITILTFTLLTRQTLYELRFRDGDKEVAALMACTSR 49
EHEC 1 MKRNPLVVCLLIICITILTFTLLTRQTLYELRFRDSDKEVAALMACTSR 49
SHIG 1 MKHNPLVVCLLIICITILTFTLLTRQTLYELRFRDGDKEVAALMACTSR 49
EPEC 1 MKRSGGVSAHYLHYDSDIHTPDPTIALRTAVPGR 34

C

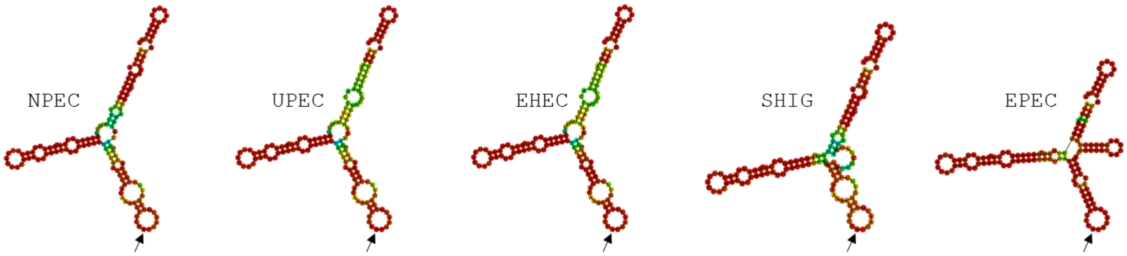

Supplementary Figure 10. A comparison between the HokB homologs in the tested strains and species analyzed in Figure 7. A. Multiple sequence alignment of *hokB* coding sequence as sequenced by Sanger sequencing and aligned to the NC_000913.3 reference genome. The genomic coordinates of *hokB's* in each tested species are: 1491922-1492071 in NC_000913.3 for non-pathogenic *E. coli* (NPEC); 152813-152962 in CP054232 for uropathogenic *E. coli* (UPEC); 2078898-2079047 in AE005174 for enterohemorrhagic *E. coli* (EHEC); 1219372-1219521 in NZ_CP064376 for *Shigella sonnei* (SHIG); and 1605308-1605452 in NC_011601 for enteropathogenic *E. coli* (EPEC). Mismatches to the reference genome (NPEC) are marked in red; the five-base deletion is marked with a black arrow; start and stop codons are in bold. **B.** Multiple sequence alignment of predicted HokB protein sequence in the tested species. Mismatches to the reference protein sequence (NPEC) are in red. **C.** Predicted RNA secondary structures of *hokB* homologs using the sequence shown in A. Black arrows mark the edited adenosine.

Supplementary Table 1. Primers used in this study.

Supplementary Table 2. Proteins identified in the mass spectrometry data.

Supplementary Table 3. Data of the non-edited and edited peptide of HokB identified in the mass spec data.

Supplementary Table 4. Quantitative mass spec data of the non-edited and edited HokB peptide used for Supplementary Figure 1.
